# Metagenomics reveals niche partitioning within the phototrophic zone of a microbial mat

**DOI:** 10.1371/journal.pone.0202792

**Published:** 2018-09-11

**Authors:** Jackson Z. Lee, R. Craig Everroad, Ulas Karaoz, Angela M. Detweiler, Jennifer Pett-Ridge, Peter K. Weber, Leslie Prufert-Bebout, Brad M. Bebout

**Affiliations:** 1 Exobiology Branch, NASA Ames Research Center, Moffett Field, CA, United States of America; 2 Bay Area Environmental Research Institute, Petaluma, CA, United States of America; 3 Earth and Environmental Sciences, Lawrence Berkeley National Laboratory, Berkeley, CA, United States of America; 4 Physical and Life Sciences Directorate, Lawrence Livermore National Laboratory, Livermore, CA, United States of America; Oklahoma State University, UNITED STATES

## Abstract

Hypersaline photosynthetic microbial mats are stratified microbial communities known for their taxonomic and metabolic diversity and strong light-driven day-night environmental gradients. In this study of the upper photosynthetic zone of hypersaline microbial mats of Elkhorn Slough, California (USA), we show how metagenome sequencing can be used to meaningfully assess microbial ecology and genetic partitioning in these complex microbial systems. Mapping of metagenome reads to the dominant *Cyanobacteria* observed in the system, *Coleofasciculus (Microcoleus) chthonoplastes*, was used to examine strain variants within these metagenomes. Highly conserved gene subsystems indicated a core genome for the species, and a number of variant genes and subsystems suggested strain level differentiation, especially for nutrient utilization and stress response. Metagenome sequence coverage binning was used to assess ecosystem partitioning of remaining microbes to both reconstruct the model organisms *in silico* and identify their ecosystem functions as well as to identify novel clades and propose their role in the biogeochemical cycling of mats. Functional gene annotation of these bins (primarily of *Proteobacteria*, *Bacteroidetes*, and *Cyanobacteria*) recapitulated the known biogeochemical functions in microbial mats using a genetic basis, and revealed significant diversity in the *Bacteroidetes*, presumably in heterotrophic cycling. This analysis also revealed evidence of putative phototrophs within the *Gemmatimonadetes* and *Gammaproteobacteria* residing in microbial mats. This study shows that metagenomic analysis can produce insights into the systems biology of microbial ecosystems from a genetic perspective and to suggest further studies of novel microbes.

## Introduction

Hypersaline microbial mats are diverse laminated assemblages of microorganisms thought to represent one of the earliest ecosystems on Earth, and are typically dominated by oxygenic phototrophic cyanobacteria [1,2]. Compact and highly structured, these mats contain microbial communities that possess great diversity at both the metabolic and phylogenetic level [3,4]. Microbial mats have been described as complete ecosystems in miniature, with relatively closed cycling of photosynthetically fixed carbon from the upper layers distributed to heterotrophic organisms in the lower layers for re-mineralization and subsequent reincorporation. Photosynthetic activity of oxygenic phototrophs during the daytime is followed by a rapid transition into anoxic conditions following sunset. Features of an active nitrogen cycle include high rates of nitrogen fixation supported by daytime photosynthetic activity or sulfide redox reactions [3,5].

Extensive biogeochemical, microbiological, and targeted molecular ecological studies have been completed on the hypersaline microbial mats of Elkhorn Slough (CA) and have identified rates of biogeochemical processes and the identities of some organisms involved. As an example, previous studies have shown that within the upper 2 mm layers of these mats, net hydrogen production is a consequence of constitutive fermentation of photosynthate to acetate by *Cyanobacteria* (dominated by the filamentous cyanobacterium *Coleofasciculus chthonoplastes*), followed by consumption of fermentation byproducts by *Desulfobacterales* and *Chloroflexi* [6–9]. Major nitrogen fixers have also been identified, and include a novel group of cyanobacteria (ESFC-1), that have been isolated and whole genome sequenced [10–12]. In many cases, identity and metabolic role of microbes have not been linked to specific biogeochemical transformations, largely due to the high diversity and novelty of the microorganisms present in these mats. A method to survey the overall diversity of novel ecosystems, such as high-throughput shotgun metagenome sequencing, could be used to assess a more complete picture of the biogeochemical cycling of these mats.

Guided by the known ecology of this ecosystem, this study aims to reconstruct the functional and microbial diversity in the phototrophic zone of microbial mats of Elkhorn Slough, CA and begin to address the knowledge gaps in the functional assignment of un-isolated clades from these mats. Underpinning this work are previous binning studies that have sought to analyze metagenomic results at the organism level rather than at the microbiome level, either to identify novel genomic diversity [13–16], novel metabolism [17], novel genetics [18,19], or ecological succession [20–22]. Co-abundance binning of metagenomic scaffolds was used to recover bins representing species, or groups of closely related organisms, in the phototrophic mat layer in order to recapitulate the organism-level functional diversity observed from physiological studies and to also suggest the ecological function of novel organisms that have yet to be isolated. During this process, subspecies diversity was noted for *C*. *chthonoplastes*. We then applied recent insights in reference-based variant analysis from metagenomes [23] to *C*. *chthonoplastes* to identify the core and varying metabolic pathways within this species.

## Methods

### Site sampling and incubation description

Samples were collected from the Elkhorn Slough estuary at 36°48’46.61” N and 121°47’4.89” W. The study was carried out on private land with the permission of the owner to conduct the study on this site. The site consists of up to 1 cm thick mats dominated by *Coleofasciculus* sp. (formerly *Microcoleus* sp.) and *Lyngbya* sp., that vary with seasonal water flows and nutrient inputs. The conditions and the mats found at this site have been documented in previous reports [6,8,10]. A single contiguous mat piece approximately 60–80 cm in diameter was harvested in a total of thirty-two 10 cm diameter acrylic cores tubes on Nov. 8, 2011 at 6 AM. Cores were sealed with rubber stoppers on the bottom, covered with clear plastic wrap and transported to NASA Ames Research Center (Moffett Field, CA) for incubation with Elkhorn Slough water in aquaria under natural light and as part of a companion study [24] split into two treatments: controls and a set treated with 30 mM molybdate to inhibit sulfate reduction. Two control and two molybdate-treated samples, collected at 1:30 PM and 1:30 AM, were selected for metagenomic sequencing. Because of the short timeframe of the study, no differences in community composition were expected amongst these samples due to the manipulations. Therefore, the four metagenomes were treated as replicates.

### Nucleic acid extraction

Nucleic acid extraction was conducted using a total RNA/DNA approach consisting of a phenol-chloroform method [10] in combination with the RNeasy Mini Elute Cleanup Kit and QIamp DNA Mini Kit (Qiagen, Hilden, Germany). A rotor-stator homogenizer (Tissue-master, Omni International, Kennesaw, GA, USA) was first washed successively with 70% EtOH, RNase-away (Sigma, St. Louis, MO, USA), and RNase-free H_2_O. The top 2 mm of the microbial mat was excised using a sterile razor blade and homogenized for 30 seconds on the lowest setting in 0.5 ml RLT buffer mix (10 ml RLT buffer (RNEasy Plus Mini kit, Qiagen) and 100 ul β-mercaptoethanol) in a 2 ml bead beating tube (0.5 mm zirconium beads). A FastPrep bead beater (MP Biomedicals, Santa Ana, CA, USA) was used for 40 seconds at setting “6.0”. Samples were spun for 1 minute at 8,000 x *g* (rcf) and the supernatant transferred to new 2 ml tubes. DNA was isolated by adding an equal volume of phenol-chloroform (basic) and vortexing for 10 seconds, incubating for 5 minutes at room temperature, and spinning for 5 minutes at 8,000 x *g* (rcf). The aqueous phase was transferred to a new tube on ice. An equal volume of 100% ethanol was added to eluate and vortexed for 10 seconds. 700 ul of supernatant/ethanol mix was added to QIAmp spin column (QIAamp DNA mini prep kit, Qiagen) and processed according to manufacturer’s instructions. Whole genome shotgun metagenomic sequencing was completed at the Joint Genome Institute (JGI) on an Illumina HiSeq 2000 platform [24].

### Read preprocessing, assembly, and annotation

Metagenomic bioinformatics methods are summarized here but are described in detail in the S3 File. 150 bp paired end reads were quality trimmed using Trimmomatic [25] and sequences from metagenomes of microbial mat samples were pooled and assembled (Ray-Meta) [26] three times using different optimal assembly word sizes, followed by Prodigal open reading frame (ORF) calling [27] and annotation by MG-RAST [28]. Reads of each metagenome were then mapped to the resulting co-assemblies to calculate read coverage. Preliminary results suggested that larger scaffolds harbored strong phylogenetic signal (S3 Fig), so these scaffolds were used for recruiting clusters representing bins from the metagenomes. Both sample coverage and %GC content were used as features for binning. First, a log transform of coverage was combined with %GC and manually scaled. Principal component analysis (PCA) of the largest three components was followed by Density-Based Spatial Clustering of Applications with Noise (DBSCAN) [29] clustering of scaffolds > 5,000 bp. These clusters were used as the basis for training data for support vector machine (SVM) classification [30] of the remaining scaffolds to obtain final bins (S4–S6 Figs). The binning process was tuned by maximizing single copy essential gene membership and minimizing gene copy duplication. This was repeated for the 3 assemblies (performed at different word sizes) and the best corresponding bin from each assembly was extracted and pooled with background and unbinned scaffolds from the k = 29 assembly. Binning procedure and quality analysis were based on analysis of ~100 essential single copy genes [31,32]. Annotations of bins were searched using a custom procedure to capture as much as possible from annotation information from EC, KEGG, SEED and several other annotations systems for analysis.

### Read mapping and variant analysis

The complete metagenomic dataset from 4 samples were pooled and mapped to the *C*. *chthonoplastes* PCC 7420 [33] genome (GCA_000155555.1 ASM15555v1, JCVI) using Bowtie2 and single nucleotide polymorphisms (SNPs) called with FreeBayes [34]. Detailed methods are available in S3 File. Variants were summarized by gene and subsystems (PATRIC) and a score developed based on the accumulation of variants in subsystems to estimate the level of genetic variation in each subsystem as compared to genome-wide variations (Eq 1).

$$Score_{g}=10^{3}\sum_{n=1,2,3,4}C_{n}\left(\frac{V_{g,n}}{V_{G,n}}-\frac{V_{g,all}}{V_{G,all}}\right)$$ (1)

Where: (*g*) is variation density for a gene set in (for all gene sets G) and V_g,n_ is the number of genes in set *g* that have variation n-1 to n (e.g. [0–1%), [1%-2%) etc.) and C_n_ is a weighting coefficient (here, C_n_ = 1, unweighted). Variations were scaled by 1,000 for readability. Positive scores indicated more genes with variation in a subsystem than the genome-wide average and negative scores indicated fewer genes with variation than the genome-wide average.

### Data and repository archival

Data and code from this study were archived to several locations. JGI sequences were archived on the JGI IMG server under Project ID 1081546–1081548, 1000633. Ray-meta assemblies, gene sequences, and gene annotation tables were archived at https://doi.org/10.5281/zenodo.1346420. All codes used on this project are available at GitHub (http://github.com/leejz/). Three-dimensional interactive ShinyRGL visualizations of galaxy plots are available at https://leejz.shinyapps.io/plot3d2 (training dataset) and at https://leejz.shinyapps.io/plot3d3 (full dataset). All other relevant data are included within the paper and its Supporting Information files.

## Results and discussion

### Metagenomic binning of the photosynthetic zone of microbial mats

**Fig 1:**
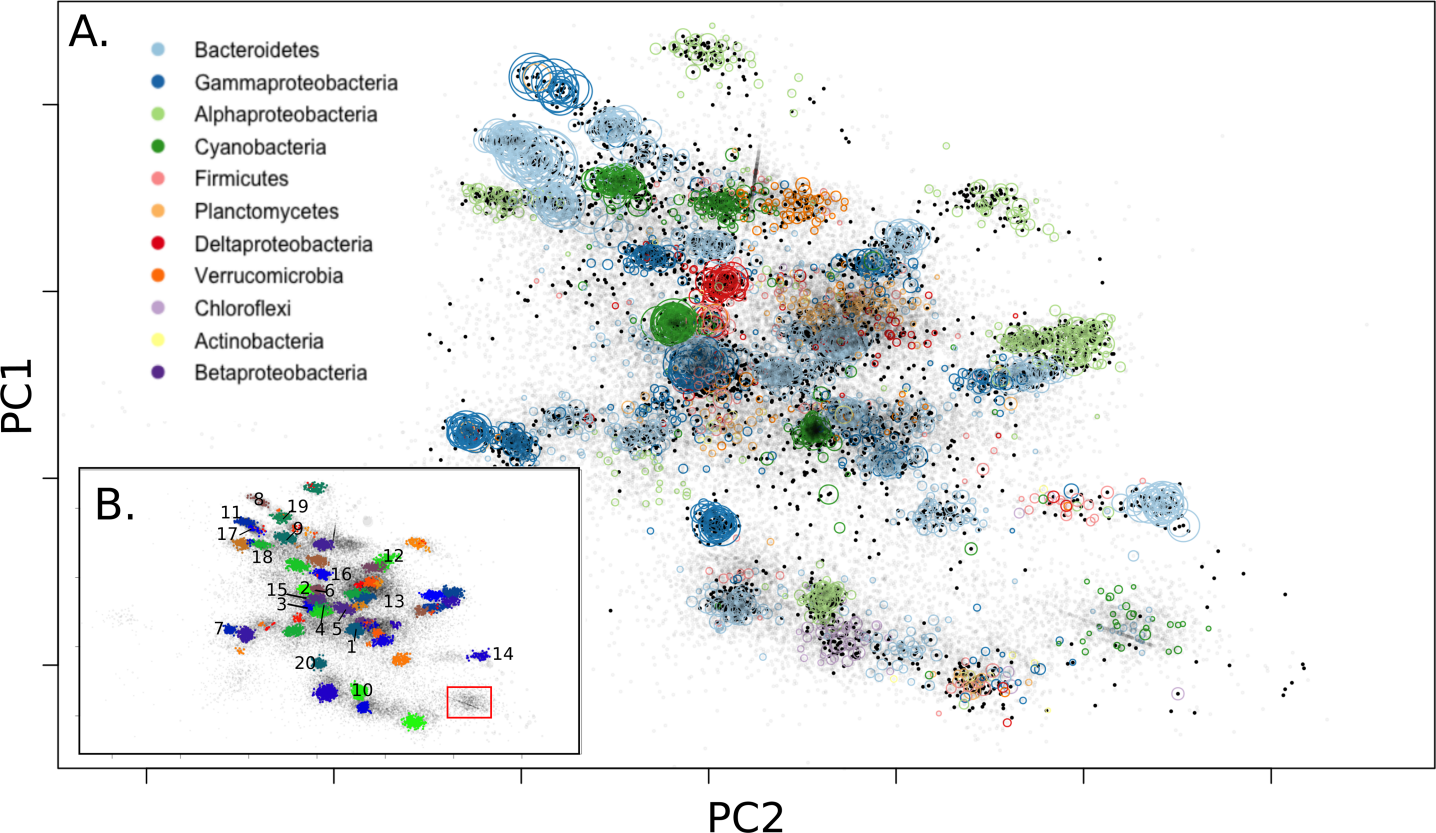
PCA galaxy chart with MG-RAST annotated abundant phyla labeled (A) and final detected bins (B). Dark dots represent the >5kbp training dataset, and light dots represent all scaffolds >1.5kbp. Colored circles represent phylum of scaffolds based on single copy essential gene classification. Size of phylum circles is proportional to scaffold size. The top two axes are shown here, but the third largest component was also used to differentiate bins. Complete bins from Table 1 are enumerated in inset (B) and Cyanobacterium ESFC-1 labeled (red box).

“Galaxy” charts of PCA components overlaid with taxonomic information from essential single copy genes found on scaffolds (Fig 1A) showed that bins could be identified from longer scaffold fragments containing these genes. When longer scaffolds were clustered, bins were clearly delineated as density-dependent clusters by the first 3 principal component axes (supplemental Shiny visualization plots). This produced more than 70 bins (Fig 1B) evaluated for completeness (fraction of essential single copy genes detected) and duplication (fraction of duplicate essential single copy genes detected) of which the top 20 bins (equivalent to those with a completeness > 80%) were selected for downstream analysis. Table 1 shows the top 20 ordered by mean coverage and S1 File shows the remainder. Two approaches to determine taxonomic affiliation were undertaken. In a more conservative approach, we selected the most common phylum annotated by Hidden Markov Model (HMM) profiles of essential single copy genes. Where taxonomy could be determined for these single copy genes, taxonomy at phylum level was concordant for each bin, with one exception (Table 1, bin 6). Our second method for taxonomy determination was to examine the most common genome annotation matched by MG-RAST for all Open Reading Frames (ORFs) in a bin. This produced a nearest genome result and the fraction of genes that matched to this nearest genome (Table 1). Of the 20 bins selected for downstream analysis, using annotations of single copy essential genes, 3 bins of *Cyanobacteria* (bins 1,2,9), 5 bins of *Gammaproteobacteria* (bins 3,4,7,8,20), 1 bin of *Alphaproteobacteria* (bin 10), 1 bin of *Deltaproteobacteria* (bin 16), 1 bin of *Firmicutes* (bin 6), and 9 bins of *Bacteroidetes* (bins 5,11,12,13,14,15,17,18,19) were annotated with near consensus at the phylum level (class level for *Proteobacteria*). These results concur with past findings of the overall types of *Proteobacteria*, *Cyanobacteria*, and *Bacteroidetes* observed in Elkhorn Slough mats [6] and with previous metagenomic studies on lithifying and non-lithifying hypersaline mats [35–37]. Consensus species taxonomic annotations from ORF annotation of the most abundant two bins (bins 1, 2) suggested that the dominant *Cyanobacteria* known from these mats (*C*. *chthonoplastes*, and *Lyngbya* sp.) were captured. The L50 assembly metric for bin 1 (*C*. *chthonoplastes*) was much lower than expected (despite the fact that this taxa was the most abundant and therefore had the deepest read sampling). The taxonomy of the third most abundant bin (bin 3) suggested that this was a mat-associated purple sulfur bacteria (*Thiorhodovibrio* sp.) [38]; the remaining *Gammaproteobacteria* (bins 4,7,8,20) were poorly matched to reference genomes. Using concatenated single-copy gene alignments, the phylogeny places these bins in 2 clades related to NOR5/OM60 and *Rhodanobacter* (S7 Fig). The lone *Firmicutes* bin (bin 6) was unique in that there was no strong consensus of phylum identity among annotated essential-copy genes, and it had ORF annotations that belonged to the only sequenced member of the phylum *Gemmatimonadetes*. Single-copy gene phylogeny also places this bin within the *Gemmatimonadetes* (S8 Fig). The single bin of *Alphaproteobacteria* (bin 10) in this subset was annotated as a possible relative of *Rhodospirillum rubrum* (54% of ORFs). Many bins in this subset annotated to various *Bacteroidetes*. Several of these bins (bins 11,13,15,19) had a closest genome match to a beach sediment chemoheterotroph, *Marivirga tractuosa* DSM 4126 [39], but only one of these (bin 19) shared a large amount of similarity (96% of ORFs). One bin (bin 14) matched to *Psychroflexus torquis* ATCC 700755 [40] (80% of ORFs) derived from Antarctic ice.

**Table 1:** Summary statistics for top assembled bins. scaffolds (L50: L50 scaffold size, Total: total Mbp binned, Mean Cov: mean coverage of all scaffolds), HMM essential single copy gene completeness (ESS: Essential single copy genes, Dup: duplicated ESS, Tot: All ESS in phylum), majority HMM phylum: majority of identified taxonomy in ESS genes, and majority MG-RAST Taxon: most common genome identified in ORFs.

**Bin**	L50	Total	Mean Cov	HMM ESS	Majority HMM Phylum	Majority MG-RAST Taxon
	kbp	Mbp		Ess/Dup/Tot	Phylum (Found/All HMM genes)	Taxa (% of ORFs)
1	9.1	8.1	118.3	93/9/106	Cyanobacteria (32/32)	Coleofasciculus chthonoplastes PCC 7420 (83%)
2	55.6	6.6	25.4	99/8/106	Cyanobacteria (50/50)	Lyngbya sp. PCC 8106 (94%)
3	31.9	5.2	19.2	104/4/105	Gammaproteobacteria (26/27)	Thiorhodovibrio sp. 970 (36%)
4	33.2	3.0	17.3	93/8/105	Gammaproteobacteria (28/30)	Alkalilimnicola ehrlichii MLHE-1 (6%)
5	17.4	4.1	16.6	95/5/105	Bacteroidetes (32/32)	Anaerophaga sp. HS1 (19%)
6	13.1	3.1	17.5	91/2/104	Firmicutes (13/26)	Gemmatimonas aurantiaca T-27 (21%)
7	13.1	2.4	15.5	97/6/105	Gammaproteobacteria (33/35)	Rhodanobacter (6%)
8	102.7	2.1	14.8	88/1/105	Gammaproteobacteria (17/18)	Alkalilimnicola ehrlichii MLHE-1 (7%)
9	31.9	5.2	14.6	96/8/106	Cyanobacteria (45/45)	Fischerella (9%)
10	12.8	3.1	12.4	84/3/105	Alphaproteobacteria (16/16)	Rhodospirillum rubrum (54%)
11	56.2	5.5	11.7	97/2/105	Bacteroidetes (36/37)	Marivirga tractuosa DSM 4126 (28%)
12	21.6	2.6	11.1	90/4/105	Bacteroidetes (13/13)	Fluviicola taffensis DSM 16823 (20%)
13	40.8	5.4	10.6	101/12/105	Bacteroidetes (45/45)	Marivirga tractuosa DSM 4126 (27%)
14	22.6	2.2	9.7	91/2/105	Bacteroidetes (30/30)	Psychroflexus torquis ATCC 700755 (80%)
15	27.5	7.2	9.4	100/8/105	Bacteroidetes (41/41)	Marivirga tractuosa DSM 4126 (31%)
16	42.0	4.1	8.2	88/4/105	Deltaproteobacteria (25/26)	Desulfotalea psychrophila LSv54 (66%)
17	145.7	3.8	7.7	105/4/105	Bacteroidetes (24/25)	Lacinutrix sp. 5H-3-7-4 (36%)
18	66.5	3.7	7.4	88/5/105	Bacteroidetes (29/29)	Anaerophaga sp. HS1 (15%)
19	10.4	3.0	7.0	86/1/105	Bacteroidetes (27/27)	Marivirga tractuosa DSM 4126 (96%)
20	42.5	3.7	6.0	101/7/105	Gammaproteobacteria (25/25)	marine gamma proteobacterium HTCC2143 (9%)

A full listing of all bins identified in this study is included in S1 File with a cross-referenced table for each assembly. We note that some of the 50+ minor bins, still largely unexamined, contained annotations for possible *Chloroflexi*, *Planctomycetes*, and *Verrucomicrobia* and may prove useful to future studies of the diversity of these organisms.

Not all canonical members of microbial mats were observed, even when unbinned sequences were searched. Notably missing from this study were the chemolithotrophic sulfur bacteria (*Beggiotoa* sp.) (S1 Fig) and methanogenic Archaea [41], both of which have been observed from Elkhorn Slough microbial mats in previous studies. Deeper sequencing efforts may be required to detect enough genomic sequence to bin these rarer clades, as was also noted for ESFC-1; alternatively sampling from deeper mat depths may be required to capture these additional species.

### Functional genetic diversity of biogeochemical cycling identified using metagenomic bin annotations

**Fig 2:**
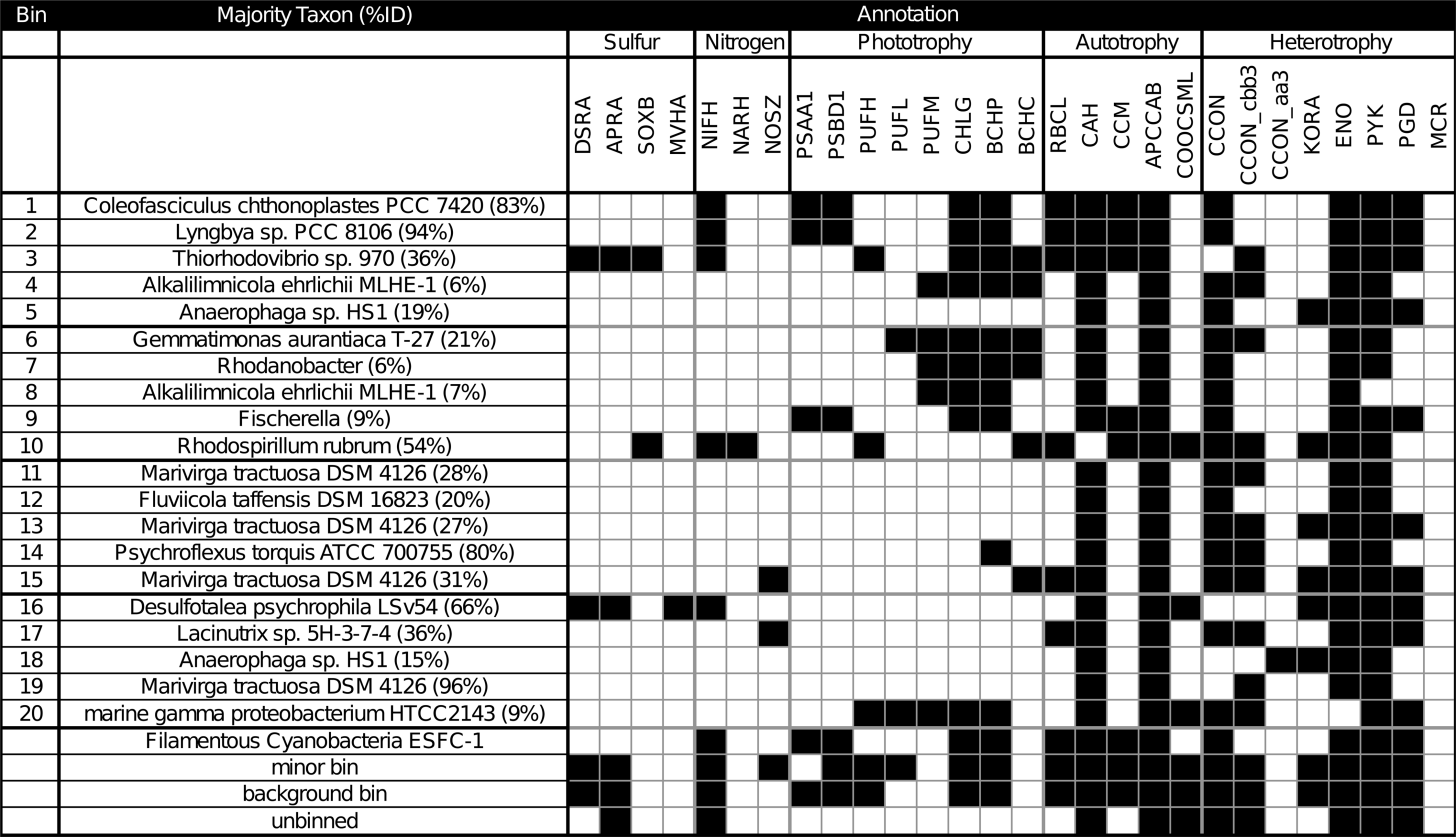
Summary table of annotated chlorophyll types and putative metabolism, one row for each major bin and one representative gene per column. Also included are annotations from Cyanobacterium ESFC-1, minor bins, background bin, or unbinned scaffolds. Each label includes a three-letter abbreviation, and subunits examined (e.g. DSRA: Dissimilatory sulfate reductase A). (Abbreviations: DSR: dissimilatory sulfite reductase, APR: adenylylsulfate reductase, SOX: sulfite oxidase, MVH: methyl viologen-reducing hydrogenase, NIF: nitrogenase, NAR: nitrate reductase, NOS: nitrous-oxide reductase, PSA: photosystem I P700 chlorophyll a apoprotein A1, PSB: photosystem II protein D1;photosystem II protein D2, PUF: photosynthetic reaction center, PSC: photosystem P840 reaction center, CHL: chlorophyll synthase;bacteriochlorophyll a synthase, BCH: bacteriochlorophyll c synthase, RBC: ribulose bisphosphate carboxylase, CAH: carbonic anhydrase, CCM: carboxysome microcompartment protein, APCC: acetyl-CoA carboxylase and/or propionyl-CoA carboxylase, COO: carbon monoxide dehydrogenase, CCON: cytochrome c oxidase, KOR: 2-oxoglutarate synthase, ENS: enolase, phosphopyruvate hydratase, PYK: pyruvate kinase, PGD: 6-phosphoglucanate dehydrogenase, MCR: methyl-coenzyme M reductase).

In this study, *de novo* metagenomic binning approaches were used to reconstruct the biogeochemical cycling divisions between different organisms of a microbial mat and to seek novel diversity previously unrevealed by reference-based annotation studies. A catalog was compiled of the pathways involved with biogeochemical cycling (C, N, S) and of the organisms involved with those pathways. Briefly, annotations of indicator genes selected from these pathways were used to identify the partitioning of biogeochemical roles across bins as well as the remaining ecosystem. Minor bins denote bins that were not in the top 20. A background recruitment bin was used for large but un-clustered scaffolds. And remaining indeterminate scaffolds were placed in an unbinned category (Fig 2). A complete list of bins, genes used in this study, gene abbreviations used in this study, and gene selection criteria can be found in S1 File.

Clear delineations between bins involved in sulfur cycling, nitrogen cycling, and carbon cycling could be observed. For example, both sulfur oxidation and sulfate reduction could be resolved. Capacity for phototrophic sulfur oxidation, represented by DSR, APR, and SOX genes for sulfur oxidation and PUF, CHL, BCH for bacterial phototrophy, was present in a bin (bin 3) representing the dominant clade of *Thiorhodovibrio* sp. Previous work had identified DSR, APR, and methyl viologen-reducing hydrogenase (MVH) as genetic indicators of sulfate reduction in mats [8,42]. Here the lone *Deltaproteobacteria* with closest relative *Desulfotalea psychrophila* LSv54 (bin 16) was annotated with these genes and confirm this previous finding. This bin also contained a suite of annotations for oxygen tolerance such as reactive oxygen scavenging indicated by rubrerythrin, thioredoxin, catalase-peroxidase, and alkyl hydroperoxide reductase, as well as direct oxygen scavenging indicated by rubredoxin, molybdopterin oxidoreductase, NADH-quinone oxidoreductase (S2 File). This supported previous studies that identified sulfate reduction in the phototrophic zone of hypersaline microbial mats and suggested that oxygen-tolerant SRBs were responsible [8,43–48]. However, isolation of such organisms has been difficult and multiple mechanisms of oxygen tolerance have been proposed [49]. Similar to previous findings, the bin we identified does not appear to contain cytochrome c oxidase or superoxide dismutase [8] but does have rubrerythrin, suggesting that this organism may scavenge oxygen using this pathway or have a syntrophic oxygen coping strategy seen in mat-derived SRBs [50,51].

A bin associated with purple non-sulfur bacteria (PNS) (bin 10) was also identified as having annotations for bacteriochlorophyll-based photoautotrophy, represented by PUF, BCH, RBC, CCM, and APCC, and carbon monoxide dehydrogenase (COO). A number of PNS bins were also noted in the minor bin dataset, annotating as relatives of *Erythrobacter* sp. (S1 File). Bin 10 also contained annotations for SOX genes and NIF genes. This supported observations of nitrogen fixation potential in purple non-sulfur bacteria in mats [5,52] but also suggests a multifaceted role (i.e. sulfate and nitrate metabolism) as seen in many PNS bacteria [53]. Altogether, nitrogen fixation was a cosmopolitan feature visible in 5 bins (1,2,3,101,6), minor bins, background bin, and un-binned data. Conversely, nitrate reduction was observed in only the PNS bacteria. De-nitrification was observed in 2 bins and may be related to the active agricultural inputs noted for Elkhorn Slough.

Lastly, bins annotated as *Bacteriodetes* were the most common in the study (bins 5,11,12,13,14,15,17,18) and contained annotations for aerobic heterotrophy, represented by cytochrome c oxidase (CCON), glycolysis genes enloase (ENO), pyruvate kinase (PYK), and 6-phosphoglucanate dehydrogenase (PG). Their multitude implied heterotrophic diversification, potentially residing in the regulation of glycan genes [54]. This diversification likely stems from high amounts of free and fixed carbon derived from spent fermentation products [9], exuded polysaccharides from *Cyanobacteria* [11], and potentially other unobserved carbon influxes such as agricultural and animal inputs. A similar phenomena is observed in saccharide-rich gut ecosystems [55,56] where abundant carbohydrate substrates have been linked to great metabolic variety among *Bacteroidetes* at the species and even strain level [57,58]. Additional emphasis on the heterotrophic niche partitioning of microbial mat ecosystems is needed to reveal which major factors such as light cycle, substrate variety, and electron donors drive heterotrophic diversification.

### Metagenomic binning predicts novel functional roles of microbes from microbial mats

Binning was also used to identify novel organisms to examine the unexplored diversity in microbial mats. In addition to the known phototrophs, we also noted a number of other bins contained phototrophic genes. Four bins (bins 4,7,8,20) annotated as novel *Gammaproteobacteria* contained bacterial phototrophic genes, represented by PUF, CHL, and BCH. Additionally, bin 6, putatively a novel *Gemmatimonadetes*, also had annotations for phototrophy. These results indicated that in microbial mats, *Gammaproteobacteria* consisted not only of the canonical sulfide-oxidizing bacteria, but also of bins with bacteriochlorophyll-containing heterotrophs containing mixotrophs similar to OM60 clades. As OM60 clades have been shown to rely on specific light, oxygen, and organic acids for mixtotrophic growth [59], this work suggests that the isolation of such bacteria would be highly dependent on mimicking specific conditions that develop during a diel cycle, such as the acetate-replete, low-oxygen, low-light initial early morning photosynthetic period of microbial mats, where their growth would be distinctive from other heterotrophs. At present, given the lack of genomic knowledge about phototrophic *Gammaproteobacteria*, accurate taxonomic assignment of these bins remains challenging.

This work also identifies a potential novel phototroph within *Gemmatimonadetes*. Based on the taxonomic identity and binning completeness we measured, our research suggests this bin represents a possible salt-tolerant variant of the recently isolated phototrophic *Gemmatimonadetes*. Sequences of taxa from freshwater lakes belonging to possible clades of phototrophic *Gemmatimonadetes* [60] bear some resemblance to this bin. Specifically, the annotations of photosystem genes in this bin appear to be derived via horizontal gene transfer from *Alphaproteobacteria*, and contain annotations for aerobic respiration genes (cytochrome c oxidase (CCO), (2-oxoglutarate synthase) KORA, enolase (ENO), pyruvate kinase (PYK), and 6-phosphoglucanate dehydrogenase (PGD), Fig 2). However, given the possibility of mis-assembly, mis-annotation, and the difficulty of inferring gene expression from genomic data, these findings must be paired with further microbial isolation, characterization, and genome sequencing.

### Bin pathway annotations of *C*. *chthonoplastes* show extensive polysaccharide production and breakdown capability compared to other mat *Cyanobacteria*

**Fig 3:**
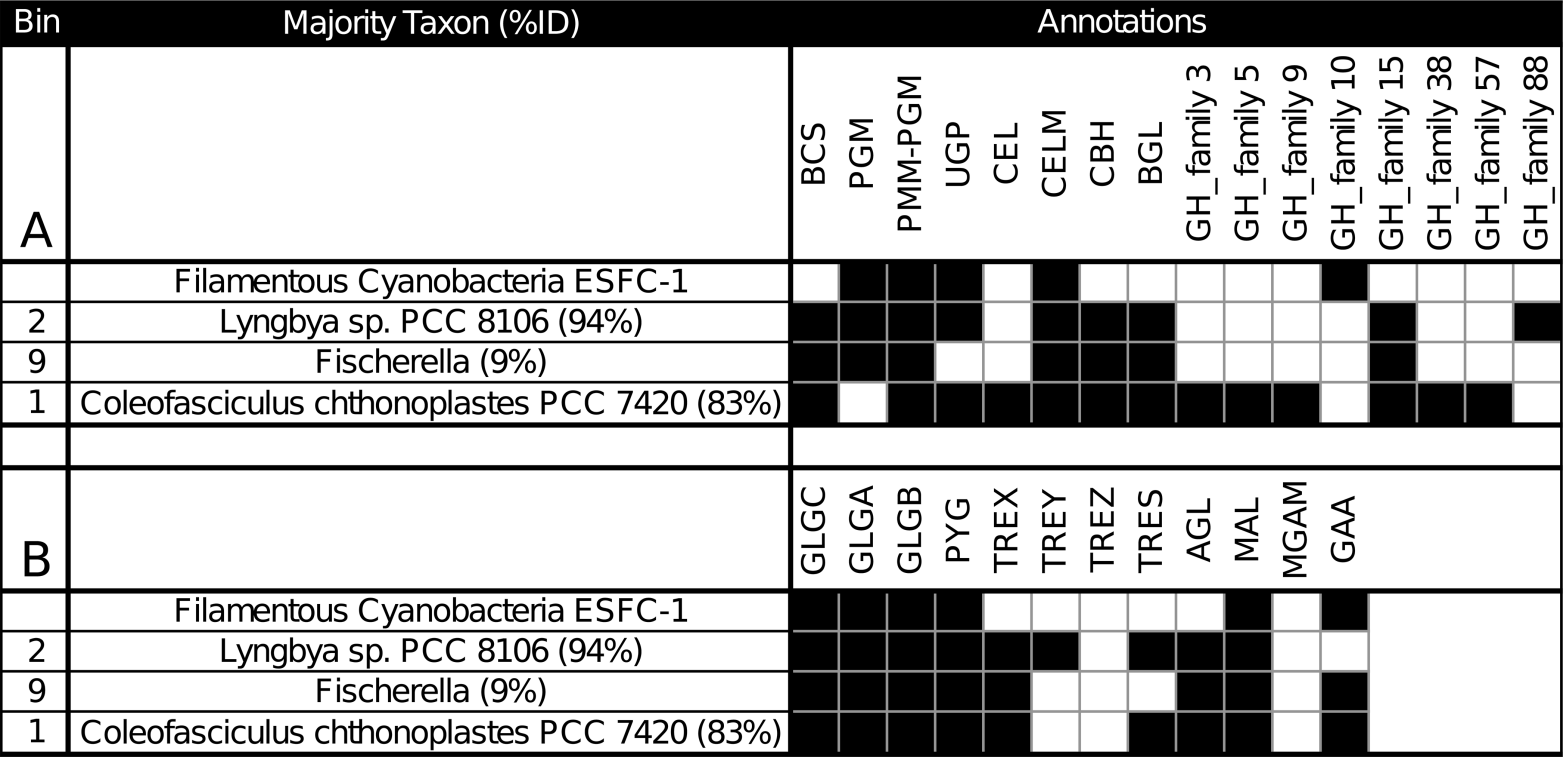
Carbohydrate regulation from *Cyanobacteria* bins and the genome of ESFC-1 indicate unique polysaccharide capability of *C*. *chthonoplastes* among mat organisms when examining cellulose production genes (A), starch production genes (B). Abbreviations: BCS: cellulose synthase, PGM: phosphoglucomutase, PMM-PGM: phosphomannomutase/phosphoglucomutase, UGP: UTP—glucose-1-phosphate uridylyltransferase, CEL: Cellulase / Endoglucanase, CELM: cellulase M, CBH: cellulose 1,4-beta-cellobiosidase, BGL: beta-glucosidase, GH: glycosyl hydrolase, GLGC: glucose-1-phosphate adenylyltransferase, GLGA: glycogen synthase, GLGB: 1,4-alpha-glucan branching enzyme, PYG: glycogen phosphorylase, TREX: glycogen operon protein, TREY: maltooligosyltrehalose synthase, TREZ: maltooligosyltrehalose trehalohydrolase, TRES: trehalose synthase, AGL: glycogen debranching enzyme, MAL: 4-alpha-glucanotransferase, MGAM: maltase-glucoamylase, GAA: alpha-glucosidase.

Binning and annotation analyses showed bins 1 and 2 closely matched to *C*. *chthonoplastes* PCC 7420 and *Lyngbya* sp. PCC 8106 respectively. A third bin (bin 9) loosely matched a *Fischerella* genome (9% of ORFs). Photosystem I/II photoautotrophy represented by PSA, PSB, CHL, RubisCO (RBC), carbonic anhydrase (CAH), carboxysome microcompartment protein (CCM), and acetyl/propionyl-CoA carboxylase (APCC) was found in these bins. To understand the metabolic differences between *Cyanobacteria* within mats, annotations from these 3 bins were combined with annotations from the draft genome of ESFC-1, a nitrogen-fixing cyanobacterium previously isolated from the same ecosystem and sequenced [10,12]. In the current study ESFC-1 did not pass binning thresholds, although it was detected in metagenomes (Fig 1B). To draw comparisons between these, the major KEGG pathways were visualized. Notable differences were observed in the carbohydrate utilization pathways (S9–S11 Fig). When annotations of ESFC-1 were included as a bin with the 3 other *Cyanobacteria* and explored in depth by searching for pathway gene terms in annotations, the *C*. *chthonoplastes* bin had a unique set of glycoside hydrolases involved in many aspects of both carbohydrate production and breakdown (GH3, GH5, GH9, GH38, GH57, Fig 3A). All bins contained annotations for several types of cellulases, cellulose synthesis genes, starch (glycogen) storage and had some involvement of maltose and sucrose synthesis and cycling (Fig 3B). A full table of all search terms used and all extracted search results are available in S1 File.

These results support recent work showing that fixed carbon polysaccharide production by *Cyanobacteria* (notably beta-glucose polysaccharides) constitutes a sizable fraction of extracellular polysaccharide [11]. The role of these beta-glucose polymers are not well understood, but may play a role in carbon storage for *Cyanobacteria* [11], or may have adhesion and anchoring functions [61,62]. These two details infer a phenotype of polysaccharide specialization that distinguishes *C*. *chthonoplastes* from other *Cyanobacteria* found in these mats.

### Reference-based variant analysis reveals core and accessory genes and pathways that differentiate *C*. *chthonoplastes* PCC 7420

**Fig 4:**
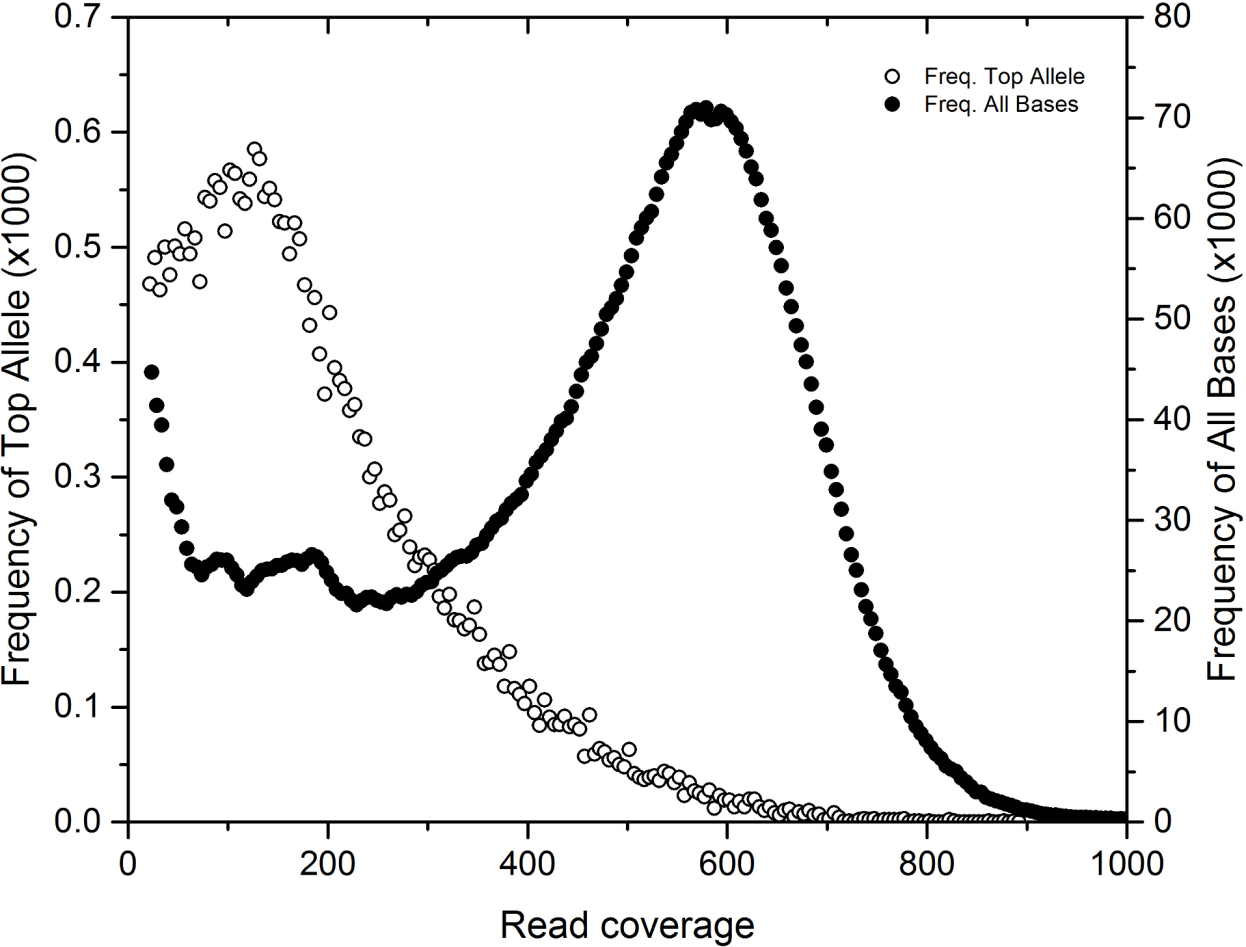
PCC7420 SNP coverage mapping histogram indicating SNP allele prevalence at lower coverage. Read coverage values for total base coverage (closed circles, right axis) and SNP dominant allele coverage (open circles, left axis).

The poor assembly statistics for the dominant bin identified in these mats (bin 1; most similar to *C*. *chthonoplastes*) suggested that genetic subpopulations of this organism confounded the assembler. We used the GenBank draft genome of the type strain PCC 7420, isolated from hypersaline microbial mats, as a mapping scaffold for metagenome reads. All variants (SNPs, indels, repeats) were inferred, but only SNPs, which were the most common variant, were retained for downstream analysis. SNPs occurred in lower coverage regions compared to the coverage distribution of the sequenced genome (Fig 4). These variants represent a rarer strain, or strains, with ~150x coverage in the sample while the main coverage distribution at ~600x coverage had fewer SNPs. SNPs from these lower coverage regions (50-200x coverage) were aggregated for each gene and each SEED subsystem. The difference between the variant density of a subsystem to the genome average was tallied for all genes in a subsystem to form a score to evaluate the relative accumulation of mutations across gene subsystems. This score weighted for subsystems with more genes. Subsystems with less variant accumulation had a negative score, and subsystems with more variant accumulation had a positive score (S12 Fig). Scores from subsystems that had the least and most accumulation are shown in Table 2. Subsystems with low variant density scores were related to photosynthesis (e.g. photosystem I/II, phycobilisome, chlorophyll), carbon fixation (e.g. cAMP, carboxysome, circadian clock), and basic cellular processes (e.g. cell division, DNA replication, ribosomal proteins, respiration, central metabolism). Comparative genomic analysis has shown that species can contain recombinant and horizontally transferred regions that allow for both core and flexible genome elements [63,64]. The reduced levels of variant accumulation in subsystems related to phototrophy, carbon fixation, and DNA repair seem consistent for the essential functioning subsystems of a photosynthetic cyanobacterium. This suggested that assessment of variability could predict conserved subsystems that represent the core genome of that organism. Conversely, subsystems associated with greater accumulation of mutations should indicate potential variation within niches.

**Table 2:** SNP alleles in subsystems indicating variable and conserved gene categories.

	Count of subsystem genes with variant rates of (upper inclusive):					Score
	all	0–1%	1–2%	2–3%	+3%	
**Most conserved subsystems**						
cAMP signaling in bacteria	87	52	11	0	0	-24.2
CO2 uptake, carboxysome	64	35	10	0	0	-15.6
Photosystem II	21	4	0	0	0	-8.9
Bacterial Cell Division	25	14	2	0	0	-8.3
Phycobilisome	18	2	0	0	0	-7.7
DNA-replication	26	16	3	0	0	-7.6
Entner-Doudoroff Pathway	20	9	1	0	0	-7.3
Ribosome SSU bacterial	16	2	0	0	0	-6.8
SigmaB stress responce regulation	23	8	3	0	0	-6.3
Chlorophyll Biosynthesis	17	10	1	0	0	-6.1
Respiratory Complex I	14	6	0	0	0	-6.0
Cyanobacterial Circadian Clock	35	21	4	1	0	-5.8
Peptidoglycan Biosynthesis	19	14	2	0	0	-5.8
Photosystem I	12	2	0	0	0	-5.1
Ton and Tol transport systems	25	16	1	1	0	-5.1
**Most variable subsystems**						
Fatty Acid Biosynthesis FASII	27	7	3	3	1	20.2
CBSS-258594.1.peg.3339 (glycotransferases)	35	16	2	4	1	20.1
Polyprenyl Diphosphate Biosynthesis	5	2	0	1	1	17.2
Isoprenoid Biosynthesis	11	3	1	1	1	15.8
DNA repair, bacterial	23	12	1	2	1	15.1
Cobalt-zinc-cadmium resistance	10	5	3	0	1	14.2
Pentose phosphate pathway	4	3	0	0	1	13.2
Carotenoids	15	10	0	1	1	13.0
Rhamnose containing glycans	12	3	4	3	0	12.8
Bacterial Chemotaxis	26	17	3	1	1	11.8
Glutamine, Glutamate, Aspartate and Asparagine Biosynthesis	13	8	2	0	1	11.7
Ammonia assimilation	12	5	1	0	1	11.0
Bacterial RNA-metabolizing Zn-dependent hydrolases	10	6	0	0	1	10.7
High affinity phosphate transporter and control of PHO regulon	13	5	1	3	0	8.9
Gene cluster associated with Met-tRNA formyltransferase	17	13	1	0	1	8.9
Maltose and Maltodextrin Utilization	14	5	5	2	0	8.7
Glutathione: Biosynthesis and gamma-glutamyl cycle	5	2	1	2	0	7.9
Sialic Acid Metabolism	5	1	1	2	0	7.9
Calvin-Benson cycle	17	6	0	0	1	7.7
Average						0.0
Median						-1.4
Standard Deviation						5.8

Examining individual genes from these subsystems with high variant density, specific genes that might indicate strain differentiation (Table 3) were regulatory proteins (P-II, kinases, cAMP proteins), membrane proteins (Co/Zn/Cd efflux, phosphate permease, O-antigen export permease, isoprenoid and carotenoid biosynthesis, fatty acid biosynthesis, and amino-sugar biosynthesis), nitrogen and amino acid cycling genes, and several transferases, and many genes related to carbohydrate modification. Additionally, environmental stress response genes, represented by chemotaxis genes, cryptochrome, and exodeoxyribonuclease, and DNA-cytosine methyltransferase, were observed. In this study, specific genes that had increased variation included a nitrogen regulatory gene involved in modulating nitrogen scavenging, a transaldolase (part of the pentose-phosphate pathway) and several glycotransferases and carbohydrate processing genes, an endonuclease involved in DNA repair, a DNA methyl-transferase related to phage immune response, a metal ion pump involved in toxicity resistance, and a number of fatty acid, carotenoid, isoprenoid biosynthesis pathway genes. In this study, subsystems and genes displaying increased variation suggest that C, N, P nutrient cycling and environmental stress response to factors such as salinity, metals, light and infection were the drivers in *C*. *chthonoplastes* genetic differentiation in mats may explain some of the ubiquity of this species across microbial mats worldwide. This is similar to a study of meltwater mats from Arctic and Antarctic ice shelves [65] suggested that coping with environmental regulation, especially in variable salinity conditions, was a primary driver of genetic functional diversity. S2 File contains the full list of subsystem and gene variant density statistics.

**Table 3:** Variant density for individual genes from subsystems with high variance score (excluding unknown genes and categories).

variants /bp	SEED Annotation	
	Product	Subsystem(s)
4.7	Nitrogen regulatory protein P-II	Ammonia assimilation
4.0	Transaldolase (EC 2.2.1.2)	Pentose phosphate pathway
3.9	Endonuclease V (EC 3.1.21.7)	DNA repair, bacterial
3.7	Probable Co/Zn/Cd efflux system membrane fusion protein	Cobalt-zinc-cadmium resistance
3.7	Octaprenyl-diphosphate synthase / Dimethylallyltransferase / Geranyltranstransferase / Geranylgeranyl pyrophosphate synthetase	Carotenoids Isoprenoid Biosynthesis Polyprenyl Diphosphate Biosynthesis
3.6	Glycosyltransferase	CBSS-258594.1.peg.3339
3.4	Maltose/maltodextrin ABC transporter, substrate binding periplasmic protein MalE	Bacterial Chemotaxis Maltose and Maltodextrin Utilization
3.4	Cytochrome d ubiquinol oxidase subunit II (EC 1.10.3.-)	Bacterial RNA-metabolizing Zn-dependent hydrolases Conserved gene cluster associated with Met-tRNA formyltransferase
3.1	3-oxoacyl-[acyl-carrier-protein] synthase, KASIII (EC 2.3.1.41)	Fatty Acid Biosynthesis FASII
3.0	Transketolase (EC 2.2.1.1)	Calvin-Benson cycle Pentose phosphate pathway
3.0	Glutamate racemase (EC 5.1.1.3)	Glutamine, Glutamate, Aspartate and Asparagine Biosynthesis
2.8	cAMP-binding proteins—catabolite gene activator and regulatory subunit of cAMP-dependent protein kinases	CBSS-258594.1.peg.3339
2.8	Glycosyltransferase	CBSS-258594.1.peg.3339
2.8	Glycosyl transferase, group 1	CBSS-258594.1.peg.3339
2.7	Phosphate transport system permease protein PstA	High affinity phosphate transporter and control of PHO regulon
2.7	cAMP-binding proteins—catabolite gene activator and regulatory subunit of cAMP-dependent protein kinases	CBSS-258594.1.peg.3339
2.6	Phosphate ABC transporter, periplasmic phosphate-binding protein PstS	High affinity phosphate transporter and control of PHO regulon
2.5	Gamma-glutamyltranspeptidase (EC 2.3.2.2)	Glutathione: Biosynthesis and gamma-glutamyl cycle
2.4	N-acetylmannosamine-6-phosphate 2-epimerase (EC 5.1.3.9)	Sialic Acid Metabolism
2.4	dTDP-4-dehydrorhamnose 3,5-epimerase (EC 5.1.3.13)	Rhamnose containing glycans
2.4	Octaprenyl-diphosphate synthase / Dimethylallyltransferase / Geranyltranstransferase / Geranylgeranyl pyrophosphate synthetase	Carotenoids Isoprenoid Biosynthesis Polyprenyl Diphosphate Biosynthesis
2.3	Glucose-1-phosphate thymidylyltransferase (EC 2.7.7.24)	Rhamnose containing glycans
2.3	Phosphoglucosamine mutase (EC 5.4.2.10)	Sialic Acid Metabolism
2.2	3-oxoacyl-[acyl-carrier-protein] synthase, KASII (EC 2.3.1.41)	Fatty Acid Biosynthesis FASII
2.2	Maltodextrin glucosidase (EC 3.2.1.20)	Maltose and Maltodextrin Utilization
2.2	Asparagine synthetase [glutamine-hydrolyzing] (EC 6.3.5.4)	Glutamine, Glutamate, Aspartate and Asparagine Biosynthesis
2.2	Phosphate transport system permease protein PstC (TC 3.A.1.7.1)	High affinity phosphate transporter and control of PHO regulon
2.2	Exodeoxyribonuclease VII large subunit (EC 3.1.11.6)	DNA repair, bacterial
2.2	Cryptochrome	DNA repair, bacterial photolyase
2.2	Gamma-glutamyltranspeptidase (EC 2.3.2.2)	Glutathione: Biosynthesis and gamma-glutamyl cycle
2.2	Chemotaxis protein CheC—inhibitor of MCP methylation	Bacterial Chemotaxis
2.1	3-oxoacyl-[acyl-carrier protein] reductase (EC 1.1.1.100)	Fatty Acid Biosynthesis FASII
2.0	O-antigen export system, permease protein	Rhamnose containing glycans
2.0	Putative sucrose phosphorylase (EC 2.4.1.7)	Maltose and Maltodextrin Utilization
2.0	DNA-cytosine methyltransferase (EC 2.1.1.37)	DNA repair, bacterial
2.0	4'-phosphopantetheinyl transferase (EC 2.7.8.-)	Fatty Acid Biosynthesis FASII

## Conclusion

In this study coverage binning was used to resolve functional genes at a genome population level and thus create a genetic basis for biogeochemical partitioning that parallels results seen from physiological and biogeochemical cycling studies. The use of reference-free binning was crucial as the majority of bins identified had only a fraction of genes matching any nearest reference genome. We also show that where reference genomes were available, a key weakness of assembly-based analysis, strain level micro-heterogeneity, can be used to generate SNP analyses to differentiate strain level differences using metagenome reads. Though the ecosystem survey in this study included a limited snapshot of a microbial mat, using only abundant organisms and key genes in metabolic pathways, we can see how the ecosystem roles of mat microbes partition the genetics within metagenomes. Our analysis also predicts a number of novel mat organisms that are as yet unidentified, including several phototrophs. We suggest that these combined reference and reference-free analysis approaches can be used to generate genetic atlases of biogeochemical cycling of novel ecosystems and direct further microbial investigation.

## Supporting information

S1 File Supplemental data 1. Contains bin annotation and indexing information, and gene pathway annotation information.
(XLSX)

S2 File Supplemental data 2. Contains genome variant information and gene annotations.
(XLSX)

S3 File Supplemental methods. Contains additional bioinformatics methods.
(PDF)

S1 Fig **Micrograph of Elkhorn Slough microbial mat showing dominant mat morphotypes A. *C*. *chthonoplastes*, B. *Lyngbya* spp., C. *Beggiotoa* spp.** Scale bar is 100 um. Photo taken by Kamil B. Stelmach and Leslie E. Prufert-Bebout.
(PDF)

S2 Fig Kmergenie plots indicating optimal word sizes for assembly (k = 29,45,63 were chosen). (PDF)

S3 Fig Phylogenetic signal relating to scaffold size and coverage. Coverage v. length for k = 29 word assembled scaffolds are shown indicating a phylogenetic signal relating to scaffold size and coverage.
(PNG)

S4 Fig k = 29 word size PCA galaxy charts and bin charts. Binning Galaxy plots (top) and bin numbers (bottom) are paired for each assembly word size.
(PDF)

S5 Fig k = 45 word size PCA galaxy charts and bin charts. Binning Galaxy plots (top) and bin numbers (bottom) are paired for each assembly word size.
(PDF)

S6 Fig k = 63 word size PCA galaxy charts and bin charts. Binning Galaxy plots (top) and bin numbers (bottom) are paired for each assembly word size.
(PDF)

S7 Fig AMPHORA2 amino acid concatenated gene phylogenies for *Gammaproteobacteria* bins. Identification of bins 4, 7,20 putative phototrophic *Gammaproteobacteria* in relation to other NOR5/OM60 clades. Only AMPHORA2 genes present in all genomes were used (23/31 genes). Bin 8 was dropped due to poor AMPHORA2 gene detection.
(PDF)

S8 Fig AMPHORA2 amino acid concatenated gene phylogenies for *Gemmatimonadetes* bins. Identification of Bin 6 within the *Gemmatimonadetes*. *Firmicutes* and *Gemmatimonadetes* reference strains were selected for comparison. Only AMPHORA2 genes present in all genomes were used (23/31 genes).
(PDF)

S9 Fig KEGG starch and sucrose metabolic pathways in bin 1, k63.7, *Coleofaciculus chthonoplastes* PCC7420 based on MG-RAST KEGG annotations. Red indicates present, blue indicates absent.
(PNG)

S10 Fig KEGG starch and sucrose metabolic pathways in bin 2, k29.1, *Lyngbya* spp. based on MG-RAST KEGG annotations. Red indicates present, blue indicates absent.
(PNG)

S11 Fig KEGG starch and sucrose metabolic pathways in bin 9, k29.13, unknown Cyanobacterium based on MG-RAST KEGG annotations. Red indicates present, blue indicates absent.
(PNG)

S12 Fig Histogram of variants per base pair of all genes in PCC 7420 (top), and histogram of subsystems scores (bottom). (PDF)

## References

[1] Awramik SM. Ancient stromatolites and microbial mats. MBL LECT BIOL. 1984;

[2] Oren A. Mats of Filamentous and Unicellular Cyanobacteria in Hypersaline Environments In: Seckbach J, Oren A, editors. Microbial Mats. Springer Netherlands; 2010 pp. 387–400. 10.1007/978-90-481-3799-2_20

[3] Canfield DE, Des Marais DJ. Biogeochemical cycles of carbon, sulfur, and free oxygen in a microbial mat. Geochim Cosmochim Acta. 1993;57: 3971–3984. 10.1016/0016-7037(93)90347-Y 11537735

[4] Ley RE, Harris JK, Wilcox J, Spear JR, Miller SR, Bebout BM, et al Unexpected Diversity and Complexity of the Guerrero Negro Hypersaline Microbial Mat. Appl Environ Microbiol. 2006;72: 3685–3695. 10.1128/AEM.72.5.3685-3695.2006 16672518 PMC1472358

[5] Bebout BM, Fitzpatrick MW, Paerl HW. Identification of the Sources of Energy for Nitrogen Fixation and Physiological Characterization of Nitrogen-Fixing Members of a Marine Microbial Mat Community. Appl Environ Microbiol. 1993;59: 1495–1503. 16348935 10.1128/aem.59.5.1495-1503.1993 PMC182109

[6] Burow LC, Woebken D, Bebout BM, McMurdie PJ, Singer SW, Pett-Ridge J, et al Hydrogen production in photosynthetic microbial mats in the Elkhorn Slough estuary, Monterey Bay. ISME J. 2012;6: 863–874. 10.1038/ismej.2011.142 22011721 PMC3309353

[7] Burow LC, Woebken D, Marshall IP, Lindquist EA, Bebout BM, Prufert-Bebout L, et al Anoxic carbon flux in photosynthetic microbial mats as revealed by metatranscriptomics. ISME J. 2013;7: 817–829. 10.1038/ismej.2012.150 23190731 PMC3603402

[8] Burow LC, Woebken D, Marshall IPG, Singer SW, Pett-Ridge J, Prufert-Bebout L, et al Identification of Desulfobacterales as primary hydrogenotrophs in a complex microbial mat community. Geobiology. 2014;12: 221–230. 10.1111/gbi.12080 24730641

[9] Lee JZ, Burow LC, Woebken D, Everroad RC, Kubo MD, Spormann AM, et al Fermentation couples Chloroflexi and sulfate-reducing bacteria to Cyanobacteria in hypersaline microbial mats. Front Microb Physiol Metab. 2014;5: 61 10.3389/fmicb.2014.00061 24616716 PMC3935151

[10] Woebken D, Burow LC, Prufert-Bebout L, Bebout BM, Hoehler TM, Pett-Ridge J, et al Identification of a novel cyanobacterial group as active diazotrophs in a coastal microbial mat using NanoSIMS analysis. ISME J. 2012;6: 1427–1439. 10.1038/ismej.2011.200 22237543 PMC3379636

[11] Stuart RK, Mayali X, Lee JZ, Craig Everroad R, Hwang M, Bebout BM, et al Cyanobacterial reuse of extracellular organic carbon in microbial mats. ISME J. 2015; 10.1038/ismej.2015.180 26495994 PMC5029224

[12] Everroad RC, Stuart RK, Bebout BM, Detweiler AM, Lee JZ, Woebken D, et al Permanent draft genome of strain ESFC-1: ecological genomics of a newly discovered lineage of filamentous diazotrophic cyanobacteria. Stand Genomic Sci. 2016;11: 53 10.1186/s40793-016-0174-6 27559430 PMC4995827

[13] Dick G, Andersson A, Baker B, Simmons S, Thomas B, Yelton AP, et al Community-wide analysis of microbial genome sequence signatures. Genome Biol. 2009;10: R85 10.1186/gb-2009-10-8-r85 19698104 PMC2745766

[14] Wrighton KC, Thomas BC, Sharon I, Miller CS, Castelle CJ, VerBerkmoes NC, et al Fermentation, Hydrogen, and Sulfur Metabolism in Multiple Uncultivated Bacterial Phyla. Science. 2012;337: 1661–1665. 10.1126/science.1224041 23019650

[15] Hanke A, Hamann E, Sharma R, Geelhoed JS, Hargesheimer T, Kraft B, et al Recoding of the stop codon UGA to glycine by a BD1-5/SN-2 bacterium and niche partitioning between Alpha- and Gammaproteobacteria in a tidal sediment microbial community naturally selected in a laboratory chemostat. Front Microbiol. 2014;5 10.3389/fmicb.2014.00231 24904545 PMC4032931

[16] Sekiguchi Y, Ohashi A, Parks DH, Yamauchi T, Tyson GW, Hugenholtz P. First genomic insights into members of a candidate bacterial phylum responsible for wastewater bulking. PeerJ. 2015;3: e740 10.7717/peerj.7400 25650158 PMC4312070

[17] Podell S, Ugalde JA, Narasingarao P, Banfield JF, Heidelberg KB, Allen EE. Assembly-Driven Community Genomics of a Hypersaline Microbial Ecosystem. PLoS ONE. 2013;8: e61692 10.1371/journal.pone.0061692 23637883 PMC3630111

[18] Hess M, Sczyrba A, Egan R, Kim T-W, Chokhawala H, Schroth G, et al Metagenomic Discovery of Biomass-Degrading Genes and Genomes from Cow Rumen. Science. 2011;331: 463–467. 10.1126/science.1200387 21273488

[19] Nielsen HB, Almeida M, Juncker AS, Rasmussen S, Li J, Sunagawa S, et al Identification and assembly of genomes and genetic elements in complex metagenomic samples without using reference genomes. Nat Biotechnol. 2014;32: 822–828. 10.1038/nbt.2939 24997787

[20] Morowitz MJ, Denef VJ, Costello EK, Thomas BC, Poroyko V, Relman DA, et al Strain-resolved community genomic analysis of gut microbial colonization in a premature infant. Proc Natl Acad Sci. 2011;108: 1128–1133. 10.1073/pnas.1010992108 21191099 PMC3024690

[21] Brown CT, Sharon I, Thomas BC, Castelle CJ, Morowitz MJ, Banfield JF. Genome resolved analysis of a premature infant gut microbial community reveals a Varibaculum cambriense genome and a shift towards fermentation-based metabolism during the third week of life. Microbiome. 2013;1: 30 10.1186/2049-2618-1-30 24451181 PMC4177395

[22] Sharon I, Morowitz MJ, Thomas BC, Costello EK, Relman DA, Banfield JF. Time series community genomics analysis reveals rapid shifts in bacterial species, strains, and phage during infant gut colonization. Genome Res. 2013;23: 111–120. 10.1101/gr.142315.112 22936250 PMC3530670

[23] Schloissnig S, Arumugam M, Sunagawa S, Mitreva M, Tap J, Zhu A, et al Genomic variation landscape of the human gut microbiome. Nature. 2013;493: 45–50. 10.1038/nature11711 23222524 PMC3536929

[24] D’haeseleer P, Lee JZ, Prufert-Bebout L, Burow LC, Detweiler AM, Weber PK, et al Metagenomic analysis of intertidal hypersaline microbial mats from Elkhorn Slough, California, grown with and without molybdate. Stand Genomic Sci. 2017;12: 67 10.1186/s40793-017-0279-6 29167704 PMC5688640

[25] Bolger AM, Lohse M, Usadel B. Trimmomatic: a flexible trimmer for Illumina sequence data. Bioinformatics. 2014;30: 2114–2120. 10.1093/bioinformatics/btu170 24695404 PMC4103590

[26] Boisvert S, Raymond F, Godzaridis É, Laviolette F, Corbeil J. Ray Meta: scalable de novo metagenome assembly and profiling. Genome Biol. 2012;13: R122 10.1186/gb-2012-13-12-r122 23259615 PMC4056372

[27] Hyatt D, Chen G-L, LoCascio PF, Land ML, Larimer FW, Hauser LJ. Prodigal: prokaryotic gene recognition and translation initiation site identification. BMC Bioinformatics. 2010;11: 119 10.1186/1471-2105-11-119 20211023 PMC2848648

[28] Meyer F, Paarmann D, D’Souza M, Olson R, Glass EM, Kubal M, et al The metagenomics RAST server–a public resource for the automatic phylogenetic and functional analysis of metagenomes. BMC Bioinformatics. 2008;9: 386 10.1186/1471-2105-9-386 18803844 PMC2563014

[29] Ester M, Kriegel H-P, Sander J, Xu X. A density-based algorithm for discovering clusters in large spatial databases with noise. AAAI Press; 1996 pp. 226–231.

[30] Chang C-C, Lin C-J. LIBSVM: A Library for Support Vector Machines. ACM Trans Intell Syst Technol. 2011;2: 27:1–27:27. 10.1145/1961189.1961199

[31] Albertsen M, Hugenholtz P, Skarshewski A, Nielsen KL, Tyson GW, Nielsen PH. Genome sequences of rare, uncultured bacteria obtained by differential coverage binning of multiple metagenomes. Nat Biotechnol. 2013;31: 533–538. 10.1038/nbt.2579 23707974

[32] Dupont CL, Rusch DB, Yooseph S, Lombardo M-J, Alexander Richter R, Valas R, et al Genomic insights to SAR86, an abundant and uncultivated marine bacterial lineage. ISME J. 2012;6: 1186–1199. 10.1038/ismej.2011.189 22170421 PMC3358033

[33] Garcia-Pichel F, Prufert-Bebout L, Muyzer G. Phenotypic and phylogenetic analyses show Microcoleus chthonoplastes to be a cosmopolitan cyanobacterium. Appl Environ Microbiol. 1996;62: 3284–3291. 8795218 10.1128/aem.62.9.3284-3291.1996 PMC168124

[34] Garrison E, Marth G. Haplotype-based variant detection from short-read sequencing. ArXiv12073907 Q-Bio. 2012; Available: http://arxiv.org/abs/1207.3907

[35] Khodadad CLM, Foster JS. Metagenomic and Metabolic Profiling of Nonlithifying and Lithifying Stromatolitic Mats of Highborne Cay, The Bahamas. PLoS ONE. 2012;7: e38229 10.1371/journal.pone.0038229 22662280 PMC3360630

[36] Harris JK, Caporaso JG, Walker JJ, Spear JR, Gold NJ, Robertson CE, et al Phylogenetic stratigraphy in the Guerrero Negro hypersaline microbial mat. ISME J. 2013;7: 50–60. 10.1038/ismej.2012.79 22832344 PMC3526174

[37] Ruvindy R, White RA Iii, Neilan BA, Burns BP. Unravelling core microbial metabolisms in the hypersaline microbial mats of Shark Bay using high-throughput metagenomics. ISME J. 2016;10: 183–196. 10.1038/ismej.2015.87 26023869 PMC4681862

[38] Overmann J, Fischer U, Pfennig N. A new purple sulfur bacterium from saline littoral sediments, Thiorhodovibrio winogradskyi gen. nov. and sp. nov. Arch Microbiol. 1992;157: 329–335. 10.1007/BF00248677

[39] Pagani I, Chertkov O, Lapidus A, Lucas S, Del Rio TG, Tice H, et al Complete genome sequence of Marivirga tractuosa type strain (H-43T). Stand Genomic Sci. 2011;4: 154–162. 10.4056/sigs.1623941 21677852 PMC3111994

[40] Bowman JP, McCammon SA, Lewis T, Skerratt JH, Brown JL, Nichols DS, et al Psychroflexus torquis gen. nov., sp. nov., a psychrophilic species from Antarctic sea ice, and reclassification of Flavobacterium gondwanense (Dobson et al. 1993) as Psychroflexus gondwanense gen. nov., comb. nov. Microbiol Read Engl. 1998;144 (Pt 6): 1601–1609. 10.1099/00221287-144-6-1601 9639931

[41] Lamarche-Gagnon G, Bebout BM. Aerobic hydrocarbon production by photosynthetic layers of a hypersaline laminated benthic microbial mat. Washington D.C.: PBI Internship Program, NASA; 2012. Planetary Biology Internship (PBI) report.

[42] Pereira IAC, Ramos AR, Grein F, Marques MC, da Silva SM, Venceslau SS. A Comparative Genomic Analysis of Energy Metabolism in Sulfate Reducing Bacteria and Archaea. Front Microbiol. 2011;2 10.3389/fmicb.2011.00069 21747791 PMC3119410

[43] Canfield DE, Des Marais DJ. Aerobic sulfate reduction in microbial mats. Science. 1991;251: 1471–1473. 11538266 10.1126/science.11538266

[44] Visscher PT, Prins RA, Gemerden H van. Rates of sulfate reduction and thiosulfate consumption in a marine microbial mat. FEMS Microbiol Lett. 1992;86: 283–293. 10.1111/j.1574-6968.1992.tb04820.x

[45] Jørgensen BB. Sulfate reduction and thiosulfate transformations in a cyanobacterial mat during a diel oxygen cycle. FEMS Microbiol Ecol. 1994;13: 303–312. 10.1111/j.1574-6941.1994.tb00077.x

[46] Teske A, Ramsing NB, Habicht K, Fukui M, Kuver J, Jorgensen BB, et al Sulfate-Reducing Bacteria and Their Activities in Cyanobacterial Mats of Solar Lake (Sinai, Egypt). Appl Environ Microbiol. 1998;64: 2943–2951. 9687455 10.1128/aem.64.8.2943-2951.1998 PMC106797

[47] Baumgartner LK, Reid RP, Dupraz C, Decho AW, Buckley DH, Spear JR, et al Sulfate reducing bacteria in microbial mats: Changing paradigms, new discoveries. Sediment Geol. 2006;185: 131–145. 10.1016/j.sedgeo.2005.12.008

[48] Fike DA, Gammon CL, Ziebis W, Orphan VJ. Micron-scale mapping of sulfur cycling across the oxycline of a cyanobacterial mat: a paired nanoSIMS and CARD-FISH approach. ISME J. 2008;2: 749–759. 10.1038/ismej.2008.39 18528418

[49] Dolla A, Fournier M, Dermoun Z. Oxygen defense in sulfate-reducing bacteria. J Biotechnol. 2006;126: 87–100. 10.1016/j.jbiotec.2006.03.041 16713001

[50] Lehmann Y, Meile L, Teuber M. Rubrerythrin from Clostridium perfringens: cloning of the gene, purification of the protein, and characterization of its superoxide dismutase function. J Bacteriol. 1996;178: 7152–7158. 10.1128/jb.178.24.7152–7158.1996 8955396 PMC178627

[51] Sigalevich P, Meshorer E, Helman Y, Cohen Y. Transition from Anaerobic to Aerobic Growth Conditions for the Sulfate-Reducing Bacterium Desulfovibrio oxyclinae Results in Flocculation. Appl Environ Microbiol. 2000;66: 5005–5012. 10.1128/AEM.66.11.5005–5012.2000 11055956 PMC92412

[52] Zehr JP, Mellon M, Braun S, Litaker W, Steppe T, Paerl HW. Diversity of heterotrophic nitrogen fixation genes in a marine cyanobacterial mat. Appl Environ Microbiol. 1995;61: 2527–2532. 16535068 10.1128/aem.61.7.2527-2532.1995 PMC1388486

[53] Yurkov V, Stackebrandt E, Holmes A, Fuerst JA, Hugenholtz P, Golecki J, et al Phylogenetic Positions of Novel Aerobic, Bacteriochlorophyll a-Containing Bacteria and Description of Roseococcus thiosulfatophilus gen. nov., sp. nov., Erythromicrobium ramosum gen. nov., sp. nov., and Erythrobacter litoralis sp. nov. Int J Syst Bacteriol. 1994;44: 427–434. 10.1099/00207713-44-3-427 7520734

[54] Pudlo NA, Urs K, Kumar SS, German JB, Mills DA, Martens EC. Symbiotic Human Gut Bacteria with Variable Metabolic Priorities for Host Mucosal Glycans. mBio. 2015;6: e01282–15. 10.1128/mBio.01282-15 26556271 PMC4659458

[55] Eckburg PB, Bik EM, Bernstein CN, Purdom E, Dethlefsen L, Sargent M, et al Diversity of the Human Intestinal Microbial Flora. Science. 2005;308: 1635–1638. 10.1126/science.1110591 15831718 PMC1395357

[56] Gill SR, Pop M, DeBoy RT, Eckburg PB, Turnbaugh PJ, Samuel BS, et al Metagenomic Analysis of the Human Distal Gut Microbiome. Science. 2006;312: 1355–1359. 10.1126/science.1124234 16741115 PMC3027896

[57] Cottrell MT, Kirchman DL. Natural Assemblages of Marine Proteobacteria and Members of the Cytophaga-Flavobacter Cluster Consuming Low- and High-Molecular-Weight Dissolved Organic Matter. Appl Environ Microbiol. 2000;66: 1692–1697. 10.1128/AEM.66.4.1692–1697.2000 10742262 PMC92043

[58] Rogers TE, Pudlo NA, Koropatkin NM, Bell JSK, Moya Balasch M, Jasker K, et al Dynamic responses of Bacteroides thetaiotaomicron during growth on glycan mixtures. Mol Microbiol. 2013;88: 876–890. 10.1111/mmi.12228 23646867 PMC3700664

[59] Spring S, Riedel T. Mixotrophic growth of bacteriochlorophyll a-containing members of the OM60/NOR5 clade of marine gammaproteobacteria is carbon-starvation independent and correlates with the type of carbon source and oxygen availability. BMC Microbiol. 2013;13: 117 10.1186/1471-2180-13-117 23705861 PMC3666943

[60] Zeng Y, Feng F, Medová H, Dean J, Koblížek M. Functional type 2 photosynthetic reaction centers found in the rare bacterial phylum Gemmatimonadetes. Proc Natl Acad Sci. 2014;111: 7795–7800. 10.1073/pnas.1400295111 24821787 PMC4040607

[61] Ross P, Mayer R, Benziman M. Cellulose biosynthesis and function in bacteria. Microbiol Rev. 1991;55: 35–58. 2030672 10.1128/mr.55.1.35-58.1991 PMC372800

[62] Römling U. Molecular biology of cellulose production in bacteria. Res Microbiol. 2002;153: 205–212. 10.1016/S0923-2508(02)01316-5 12066891

[63] Fraser C, Hanage WP, Spratt BG. Recombination and the Nature of Bacterial Speciation. Science. 2007;315: 476–480. 10.1126/science.1127573 17255503 PMC2220085

[64] Kashtan N, Roggensack SE, Rodrigue S, Thompson JW, Biller SJ, Coe A, et al Single-Cell Genomics Reveals Hundreds of Coexisting Subpopulations in Wild Prochlorococcus. Science. 2014;344: 416–420. 10.1126/science.1248575 24763590

[65] Varin T, Lovejoy C, Jungblut AD, Vincent WF, Corbeil J. Metagenomic Analysis of Stress Genes in Microbial Mat Communities from Antarctica and the High Arctic. Appl Environ Microbiol. 2012;78: 549–559. 10.1128/AEM.06354-11 22081564 PMC3255749

