# Supplementary material for: Metagenomics reveals niche partitioning within the phototrophic zone of a microbial mat
Source: PLoS One. 2018 Sep 11;13(9):e0202792. doi: 10.1371/journal.pone.0202792 (PMC6133358; doi:10.1371/journal.pone.0202792)
Supplement: S3 File — Contains additional bioinformatics methods. (PDF) [file pone.0202792.s003.pdf]

Supplemental Methods for:

Lee, JZ et al. Metagenomic binning of microbial mats reveals resource partitioning and novel diversity in mat phototrophy. PLOS ONE

Supplemental Methods

These methods supplement the methods given in the manuscript and are also paired with a bioinformatics guide online along with the code base used in this study on GitHub (http://github.com/leejz/meta-omics-scripts).

Metagenomic assembly, coverage, and binning methods

Quality filtered reads were assembly with Ray-Meta [1] on the NERSC Edison supercomputing cluster. Sequences from metagenomes of microbial mat samples were pooled together and co-assembled with Ray-Meta three times using different assembly word sizes (k=29, 45, 63). Assembly word sizes were assessed using KmerGenie [2] (S2 Fig). Each individual metagenome sample was then mapped using Bowtie2 [3] back to each assembled scaffolds to determine sample specific coverage. Prodigal [4] was then used to predict open reading frames (ORFs), and an HMM model [5] was used to find the essential single copy genes [6]. All ORFs were submitted to MG-RAST [7] for annotation using a BLAT 90% clustering protocol. The mapping process was repeated for open reading frames (ORFs) detected by Prodigal. MG-RAST custom md5 matches were quality filtered, de-replicated, and parsed to a tab-delimited ORF database of ORF name, protein annotation, ontology annotation, taxonomy, and coverage using a custom script.

Previous work examining binning has shown several successes using k-mer nucleotide frequency, %GC, read coverage, taxonomy, or a combination of these strategies as biosignatures of genomes within metagenomes. The pipeline used in

this study combines supervised learning [8], dimensionality reduction [9], and the coverage binning [6] using R and CRAN analysis packages.

As was noted in a previous study [8], preliminary results suggested that larger scaffolds harbored strong phylogenetic signal (S2A Fig), so these scaffolds were used to recruit clusters representing bins from the metagenomes. Log normalization and principle component analysis (PCA) dimensionality reduction using scaled values of %GC and differential sample coverage aided to resolve binning 'spears' seen in metagenomic data. DBScan, noted for being sensitive to cluster density and used on noisy datasets [10], was selected to cluster large (>5 kbp) scaffolds in PCA space. The remaining scaffolds >1.5 kbp were recruited using a SVM machine-learning algorithm [11] trained on the larger scaffolds (S3B Fig) and was tuned by maximizing single copy essential gene membership and minimizing gene copy duplication. This was repeated for the 3 assemblies (performed at different word sizes (k=29, 45, 63)); the best corresponding bin (maximum single copy genes, minimum duplication) from each assembly was extracted and pooled with both background and unbinned scaffolds from the k=29 assembly. Binning procedure and quality analysis were based on analysis of ~100 essential single copy genes [6,12]. These data when charted together produce informationally dense PCA graphs overlaying the clustering of scaffolds with scaffold information (taxonomy, scaffold size, etc.) which we refer to as "galaxy" plots.

Annotation search and collation

Since a number of different ontology systems and gene annotation databases were used to annotate genes, querying single annotation sets (e.g. KEGG) produced

partial results. To maximize search coverage, a customized Python regular expression search algorithm was developed. This algorithm emphasized matching KEGG and EC ontologies (e.g. K02586 and 1.18.6.1, with subunit or chain designation), but can also search for multiple text patterns in protein annotations by keyword and subunit (e.g. nitrogenase alpha chain), and protein abbreviation (e.g. nifD). The algorithm also allows for nested searches (e.g. first cytochrome c oxidase, then cbb3 subtype), as well as exclusion terms (e.g. 'precursor' proteins). Results were cross-referenced with bin annotation and written to tab-delimited files for heatmap generation in Excel. A list of genes associated with biogeochemical cycling (sulfur metabolism, nitrogen metabolism, phototrophy, autotrophy, and hetrotrophy) and with starch utilization were determined and used to query annotation records.

Phylogenetic analysis

Due to taxonomic ambiguity and novelty of bins, some bins were subjected to follow-on phylogenetic analysis. The AMPHORA2 pipeline [13] was used to extract amino acid sequences of highly conserved single-copy genes from scaffolds belonging to these bins and related PATRIC genomes for comparison. These genes were aligned individually with MUSCLE [14] and then filtered for positions with >10% gaps in the alignment. Next, RAxML [15] was used to construct phylogenetic trees (model: PROTCATBLOSUM62, with 100 rapid bootstrap trees) of bins and reference genomes. The best model was labeled with bootstrap annotation and used for nearest-neighbor identification of bins of interest.

Read mapping and variant analysis

Recent studies have examined the possibility of using variant callers typically seen in human genomic variant analysis for the detection of strain variation across genomes [16] and for differing populations of *Bacteria* in the human gut microbiome [17]. We used these insights and approach to call the coverage and density of variants in genes and in subsystems that differed from the mapping reference. The complete metagenomic dataset from the selected four mat samples were pooled and mapped to the *C. chthonoplastes* PCC 7420 [18,19] genome using Bowtie2 with default settings. This strain was first isolated by Waterbury as *Microcoleus chthonoplastes* in 1974 and with genome sequencing by JCVI (GCA_000155555.1, ASM15555v1). Single nucleotide polymorphisms (SNPs) were called with FreeBayes using haploid continuous pooled variant calling settings [20]. Variants were filtered for poorly called variants and selected for SNPs using Samtools and BCFtools [21,22]. Bedtools [23] was used to count the number of variants per gene in the PCC 7420 genome. Variants were summarized by gene and SEED subsystems using PATRIC annotations [24] cross-referenced with NCBI annotations (with a cutoff >70% annotation overlap using Bedtools). SNPs were then filtered for coverage between 50-200 reads and the ratio of SNPs per all bases in a gene (SNP density) was calculated. These gene SNP density values were matched to subsystem ontology. Each gene was binned by SNP density into 4 groups, (0-1%, 1-2%, 2-3%, 3%+) variants / gene length. A score was developed based on the aggregation of gene variant density in subsystems to estimate the level of genetic variation in each subsystem as compared to genome-wide variation levels (Equation 1)

$$Score_g = 10^3 \sum_{n=1,2,3,4} C_n \left(\frac{V_{g,n}}{V_{G,n}} - \frac{V_{g,all}}{V_{G,all}}\right) \quad (1)$$

where: g is a subsystem gene set from all gene sets G, n is the variant density bin described above, and $V_{g,n}$ is the number of genes in set g also in bin n, and C_n is a weighting coefficient for each bin (here, C_n= 1, unweighted). A variation ratio of $V_{g,n}$ to number of genes in n for G ($V_{G,n}$) was calculated and offset by the variation ratio of *all* gene bins in subsystem g and then summed for each bin. The result was scaled by an arbitrary factor of 1,000 for convenience to generate a final score for each gene set g. Positive scores indicated more genes with variation in a subsystem than the genome-wide average, negative scores indicated fewer genes with variation than the genome-wide average.

References

1. Boisvert S, Raymond F, Godzaridis É, Laviolette F, Corbeil J. Ray Meta: scalable de novo metagenome assembly and profiling. Genome Biol. 2012;13: R122. doi:10.1186/gb-2012-13-12-r122

2. Chikhi R, Medvedev P. Informed and automated k-mer size selection for genome assembly. Bioinformatics. 2014;30: 31–37. doi:10.1093/bioinformatics/btt310

3. Langmead B, Salzberg SL. Fast gapped-read alignment with Bowtie 2. Nat Methods. 2012;9: 357–359. doi:10.1038/nmeth.1923

4. Hyatt D, Chen G-L, LoCascio PF, Land ML, Larimer FW, Hauser LJ. Prodigal: prokaryotic gene recognition and translation initiation site identification. BMC Bioinformatics. 2010;11: 119. doi:10.1186/1471-2105-11-119

5. Eddy SR. Accelerated Profile HMM Searches. PLoS Comput Biol. 2011;7: e1002195. doi:10.1371/journal.pcbi.1002195

6. Albertsen M, Hugenholtz P, Skarshewski A, Nielsen KL, Tyson GW, Nielsen PH. Genome sequences of rare, uncultured bacteria obtained by differential coverage binning of multiple metagenomes. Nat Biotechnol. 2013;31: 533–538. doi:10.1038/nbt.2579

7. Meyer F, Paarmann D, D'Souza M, Olson R, Glass EM, Kubal M, et al. The metagenomics RAST server – a public resource for the automatic phylogenetic and functional analysis of metagenomes. BMC Bioinformatics. 2008;9: 386. doi:10.1186/1471-2105-9-386

8. Dick G, Andersson A, Baker B, Simmons S, Thomas B, Yelton AP, et al. Community-wide analysis of microbial genome sequence signatures. Genome Biol. 2009;10: R85. doi:10.1186/gb-2009-10-8-r85

9. Woyke T, Teeling H, Ivanova NN, Huntemann M, Richter M, Gloeckner FO, et al. Symbiosis insights through metagenomic analysis of a microbial consortium. Nature. 2006;443: 950–955. doi:10.1038/nature05192

10. Ester M, Kriegel H-P, Sander J, Xu X. A density-based algorithm for discovering clusters in large spatial databases with noise. AAAI Press; 1996. pp. 226–231.

11. Chang C-C, Lin C-J. LIBSVM: A Library for Support Vector Machines. ACM Trans Intell Syst Technol. 2011;2: 27:1–27:27. doi:10.1145/1961189.1961199

12. Dupont CL, Rusch DB, Yooseph S, Lombardo M-J, Alexander Richter R, Valas R, et al. Genomic insights to SAR86, an abundant and uncultivated marine bacterial lineage. ISME J. 2012;6: 1186–1199. doi:10.1038/ismej.2011.189

13. Wu M, Scott AJ. Phylogenomic analysis of bacterial and archaeal sequences with AMPHORA2. Bioinforma Oxf Engl. 2012;28: 1033–1034. doi:10.1093/bioinformatics/bts079

14. Edgar RC. MUSCLE: multiple sequence alignment with high accuracy and high throughput. Nucleic Acids Res. 2004;32: 1792–1797. doi:10.1093/nar/gkh340

15. Stamatakis A. RAxML version 8: a tool for phylogenetic analysis and post-analysis of large phylogenies. Bioinformatics. 2014;30: 1312–1313. doi:10.1093/bioinformatics/btu033

16. He M, Sebaihia M, Lawley TD, Stabler RA, Dawson LF, Martin MJ, et al. Evolutionary dynamics of Clostridium difficile over short and long time scales. Proc Natl Acad Sci. 2010;107: 7527–7532. doi:10.1073/pnas.0914322107

17. Schloissnig S, Arumugam M, Sunagawa S, Mitreva M, Tap J, Zhu A, et al. Genomic variation landscape of the human gut microbiome. Nature. 2013;493: 45–50. doi:10.1038/nature11711

18. Rippka R, Deruelles J, Waterbury JB, Herdman M, Stanier RY. Generic Assignments, Strain Histories and Properties of Pure Cultures of Cyanobacteria. Microbiology. 1979;111: 1–61. doi:10.1099/00221287-111-1-1

19. Garcia-Pichel F, Prufert-Bebout L, Muyzer G. Phenotypic and phylogenetic analyses show Microcoleus chthonoplastes to be a cosmopolitan cyanobacterium. Appl Environ Microbiol. 1996;62: 3284–3291.

20. Garrison E, Marth G. Haplotype-based variant detection from short-read sequencing. ArXiv12073907 Q-Bio. 2012; Available: http://arxiv.org/abs/1207.3907

21. Li H, Handsaker B, Wysoker A, Fennell T, Ruan J, Homer N, et al. The Sequence Alignment/Map format and SAMtools. Bioinformatics. 2009;25: 2078–2079. doi:10.1093/bioinformatics/btp352

22. Danecek P, Auton A, Abecasis G, Albers CA, Banks E, DePristo MA, et al. The variant call format and VCFtools. Bioinformatics. 2011;27: 2156–2158. doi:10.1093/bioinformatics/btr330

23. Quinlan AR, Hall IM. BEDTools: a flexible suite of utilities for comparing genomic features. Bioinforma Oxf Engl. 2010;26: 841–842. doi:10.1093/bioinformatics/btq033

24. Wattam AR, Abraham D, Dalay O, Disz TL, Driscoll T, Gabbard JL, et al. PATRIC, the bacterial bioinformatics database and analysis resource. Nucleic Acids Res. 2013; gkt1099. doi:10.1093/nar/gkt1099
