# Supplementary figures and images for: Metagenomics reveals niche partitioning within the phototrophic zone of a microbial mat
Source: PLoS One. 2018 Sep 11;13(9):e0202792. doi: 10.1371/journal.pone.0202792 (PMC6133358; doi:10.1371/journal.pone.0202792)

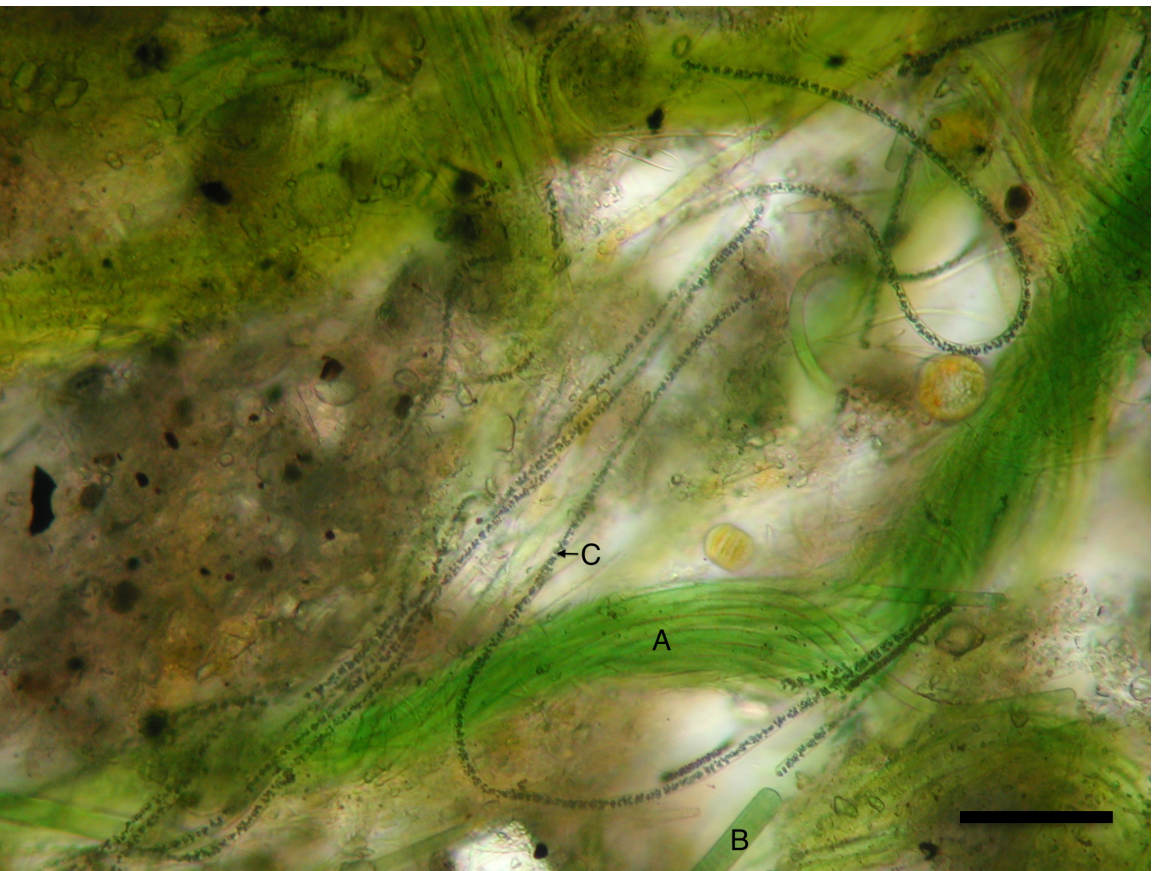

Supplement: S1 Fig — Micrograph of Elkhorn Slough microbial mat showing dominant mat morphotypes A. C. chthonoplastes, B. Lyngbya spp., C. Beggiotoa spp. Scale bar is 100 um. Photo taken by Kamil B. Stelmach and Leslie E. Prufert-Bebout. (PDF) [file pone.0202792.s004.pdf]

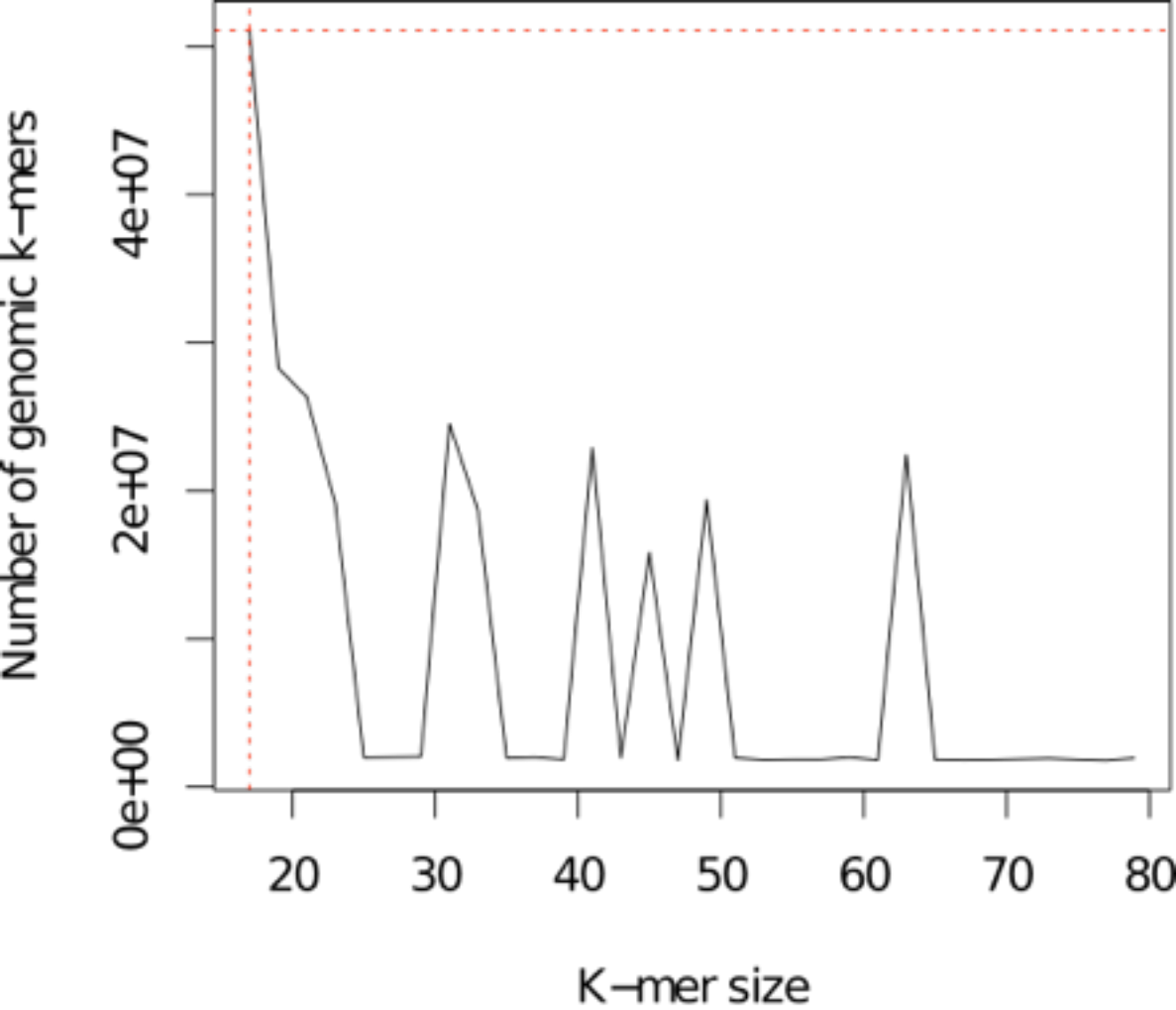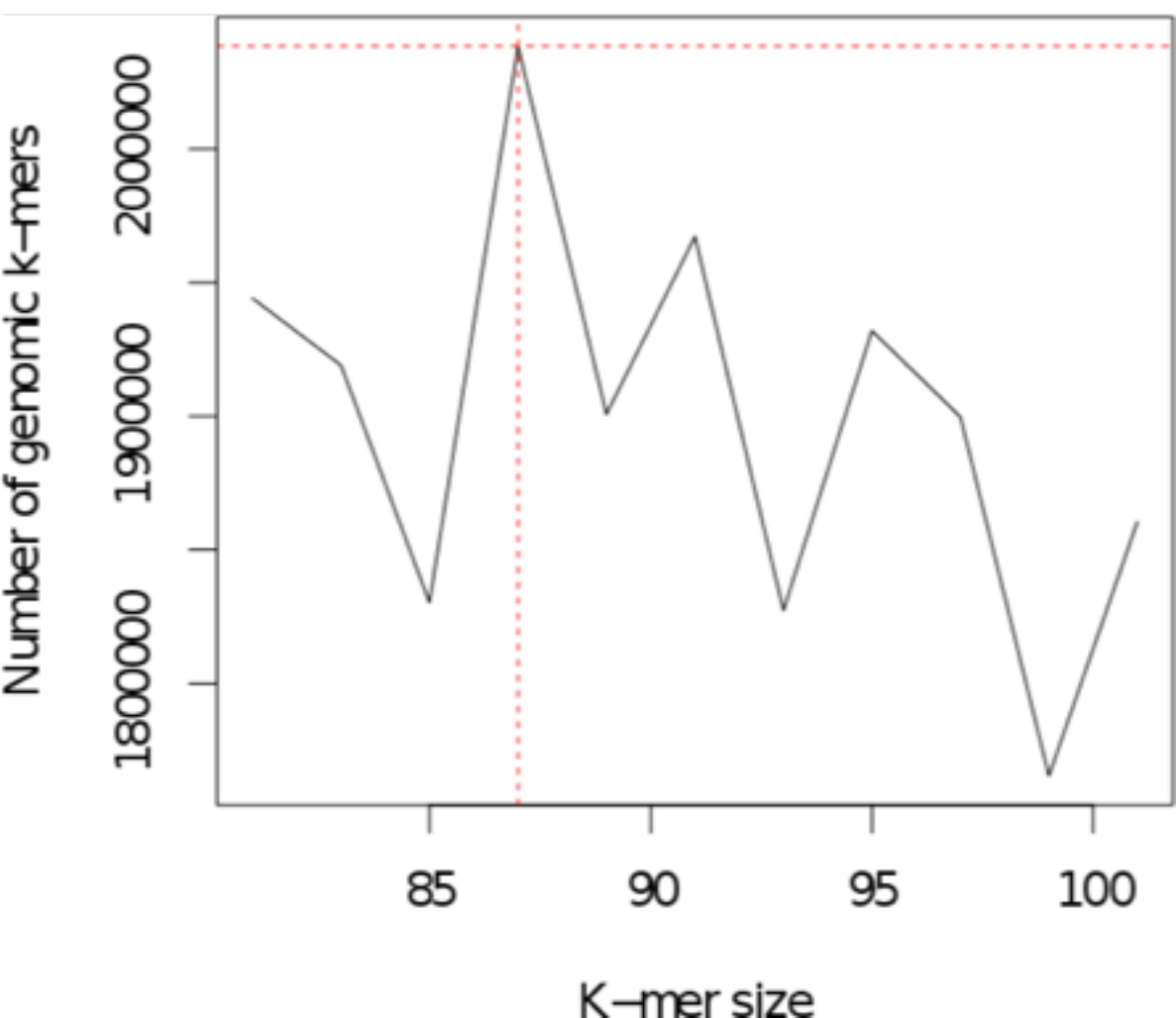

Supplement: S2 Fig — (PDF) [file pone.0202792.s005.pdf]

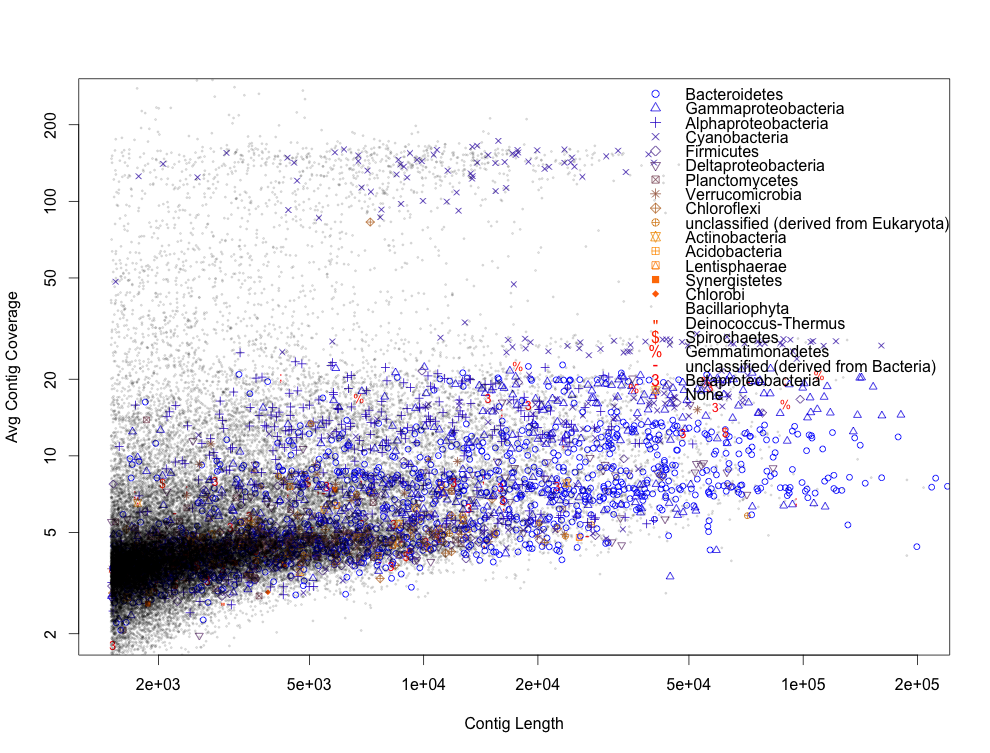

Supplement: S3 Fig — Coverage v. length for k = 29 word assembled scaffolds are shown indicating a phylogenetic signal relating to scaffold size and coverage. (PNG) [file pone.0202792.s006.png]

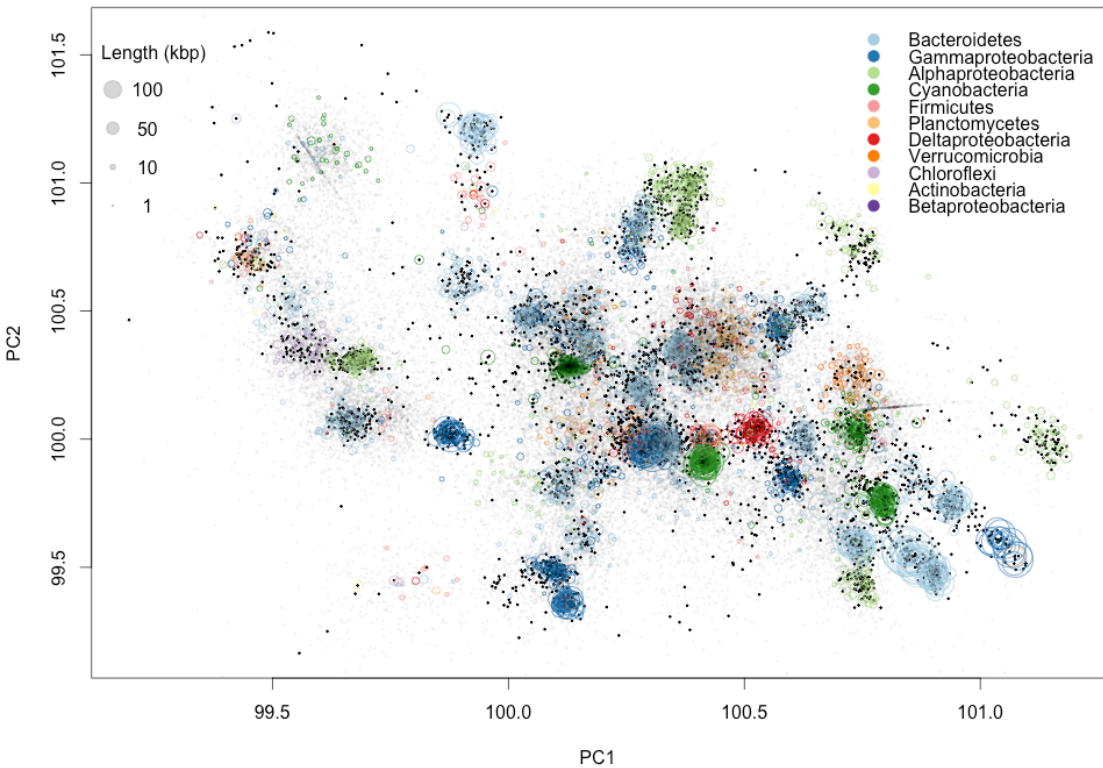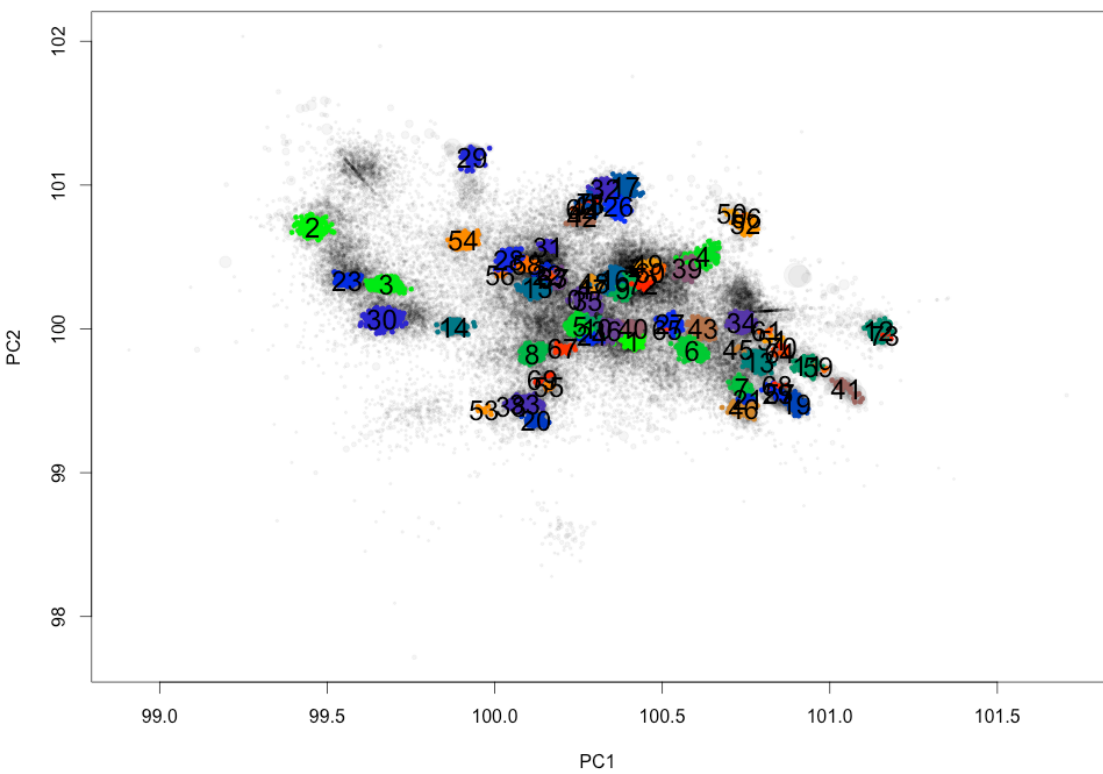

Supplement: S4 Fig — Binning Galaxy plots (top) and bin numbers (bottom) are paired for each assembly word size. (PDF) [file pone.0202792.s007.pdf]

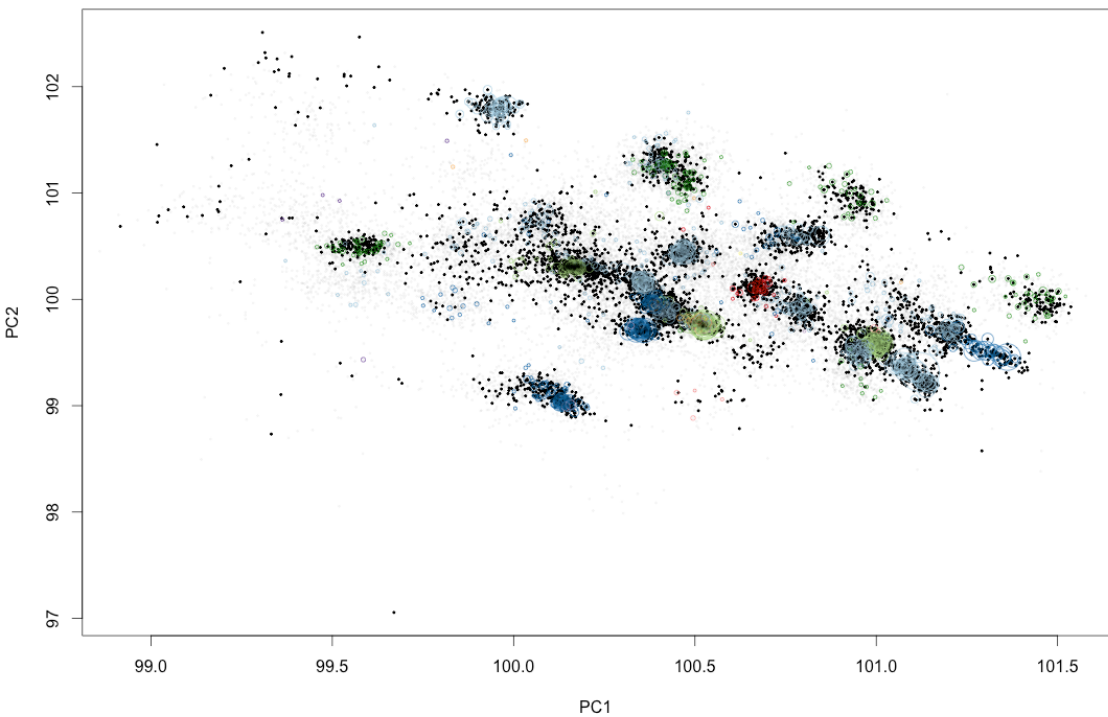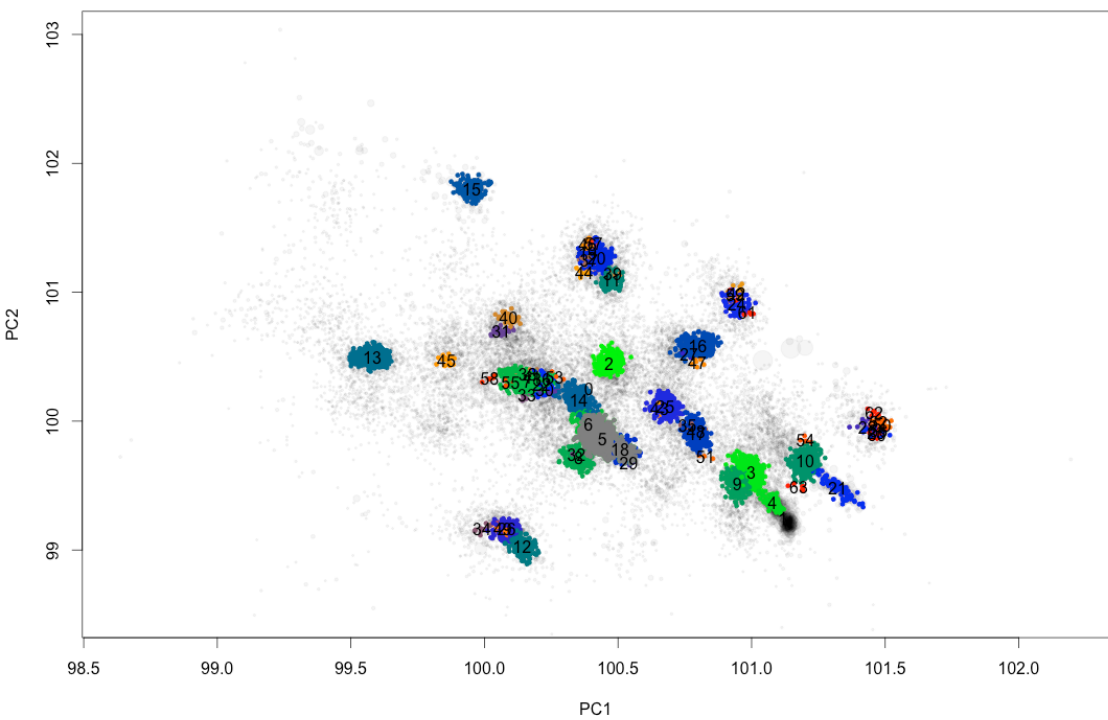

Supplement: S6 Fig — Binning Galaxy plots (top) and bin numbers (bottom) are paired for each assembly word size. (PDF) [file pone.0202792.s009.pdf]

**A.**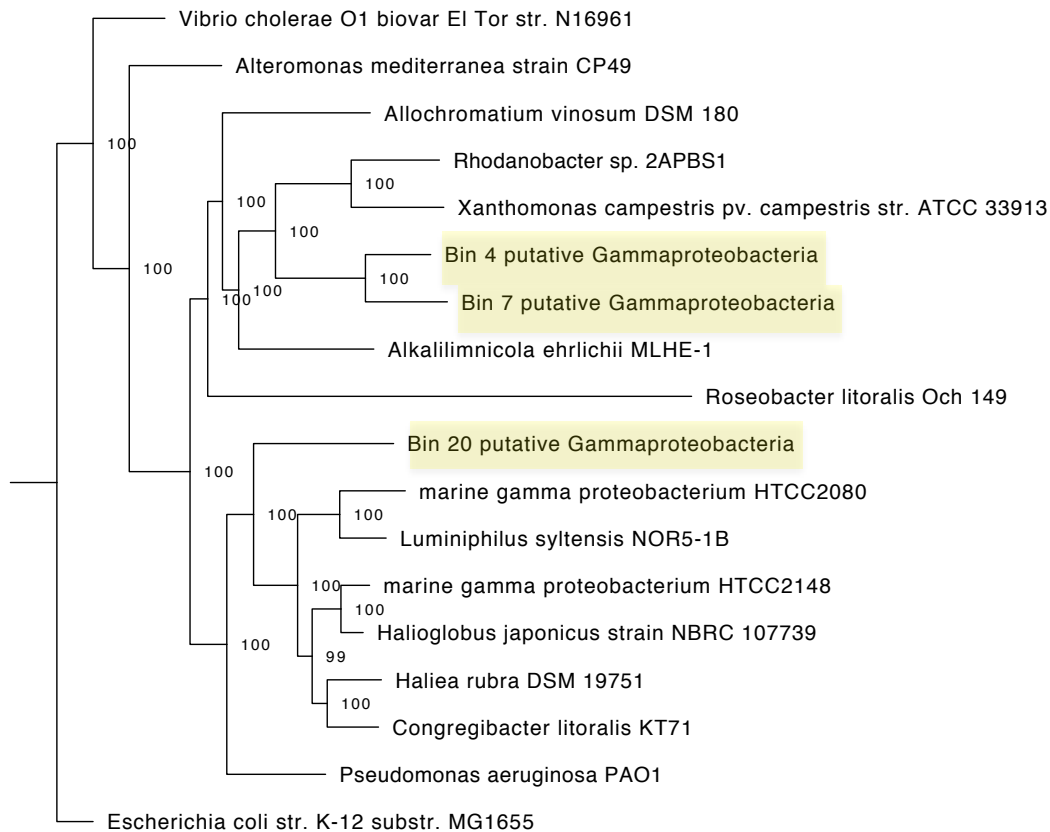

0.2

Supplement: S7 Fig — Identification of bins 4, 7,20 putative phototrophic Gammaproteobacteria in relation to other NOR5/OM60 clades. Only AMPHORA2 genes present in all genomes were used (23/31 genes). Bin 8 was dropped due to poor AMPHORA2 gene detection. (PDF) [file pone.0202792.s010.pdf]

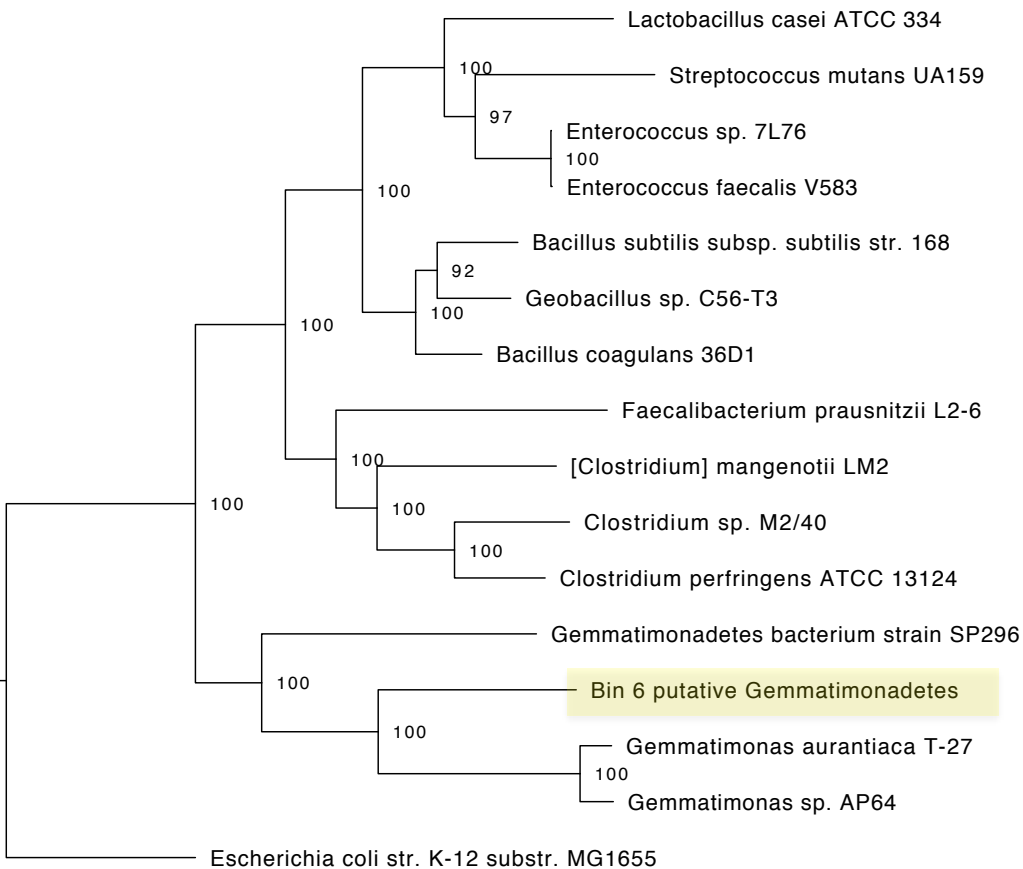

0.1

Supplement: S8 Fig — Identification of Bin 6 within the Gemmatimonadetes. Firmicutes and Gemmatimonadetes reference strains were selected for comparison. Only AMPHORA2 genes present in all genomes were used (23/31 genes). (PDF) [file pone.0202792.s011.pdf]

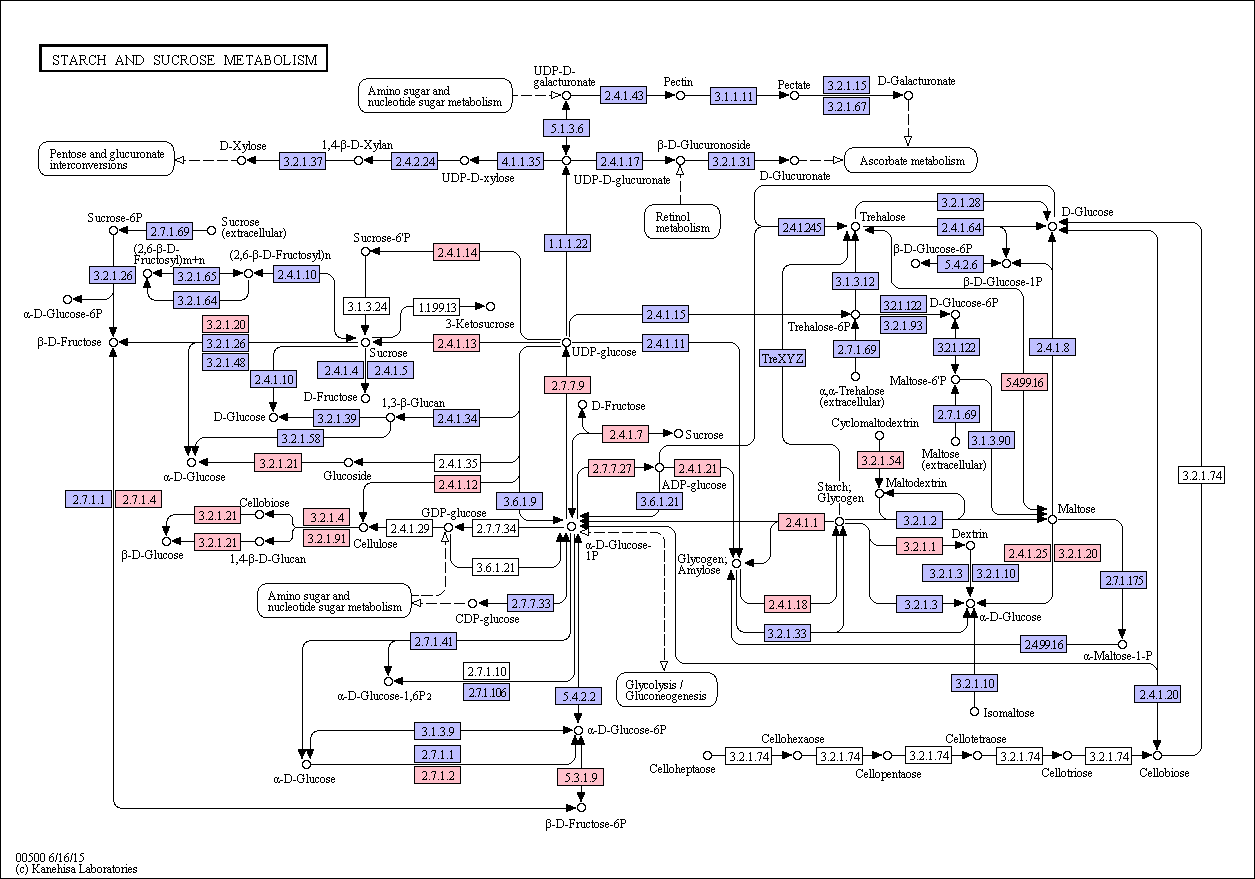

Supplement: S9 Fig — Red indicates present, blue indicates absent. (PNG) [file pone.0202792.s012.png]

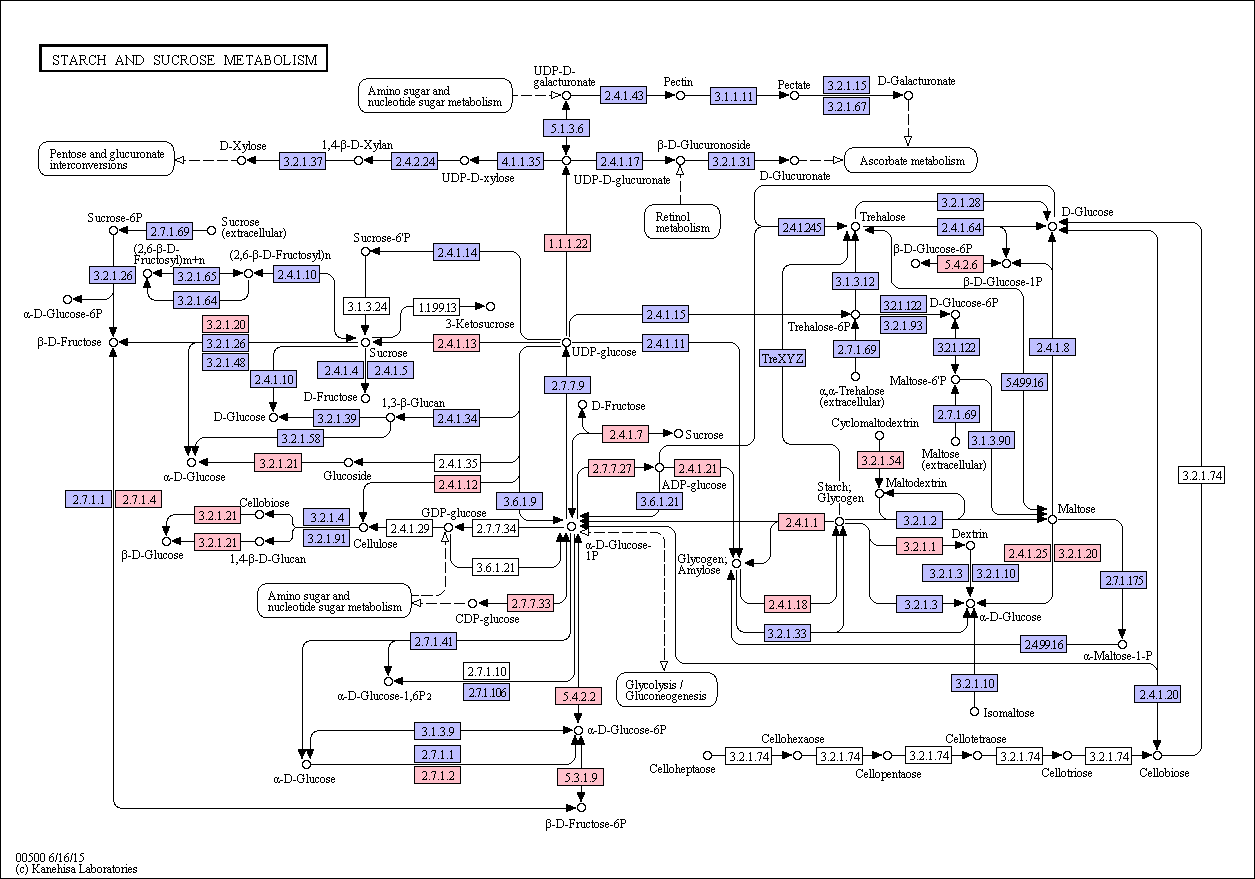

Supplement: S10 Fig — Red indicates present, blue indicates absent. (PNG) [file pone.0202792.s013.png]

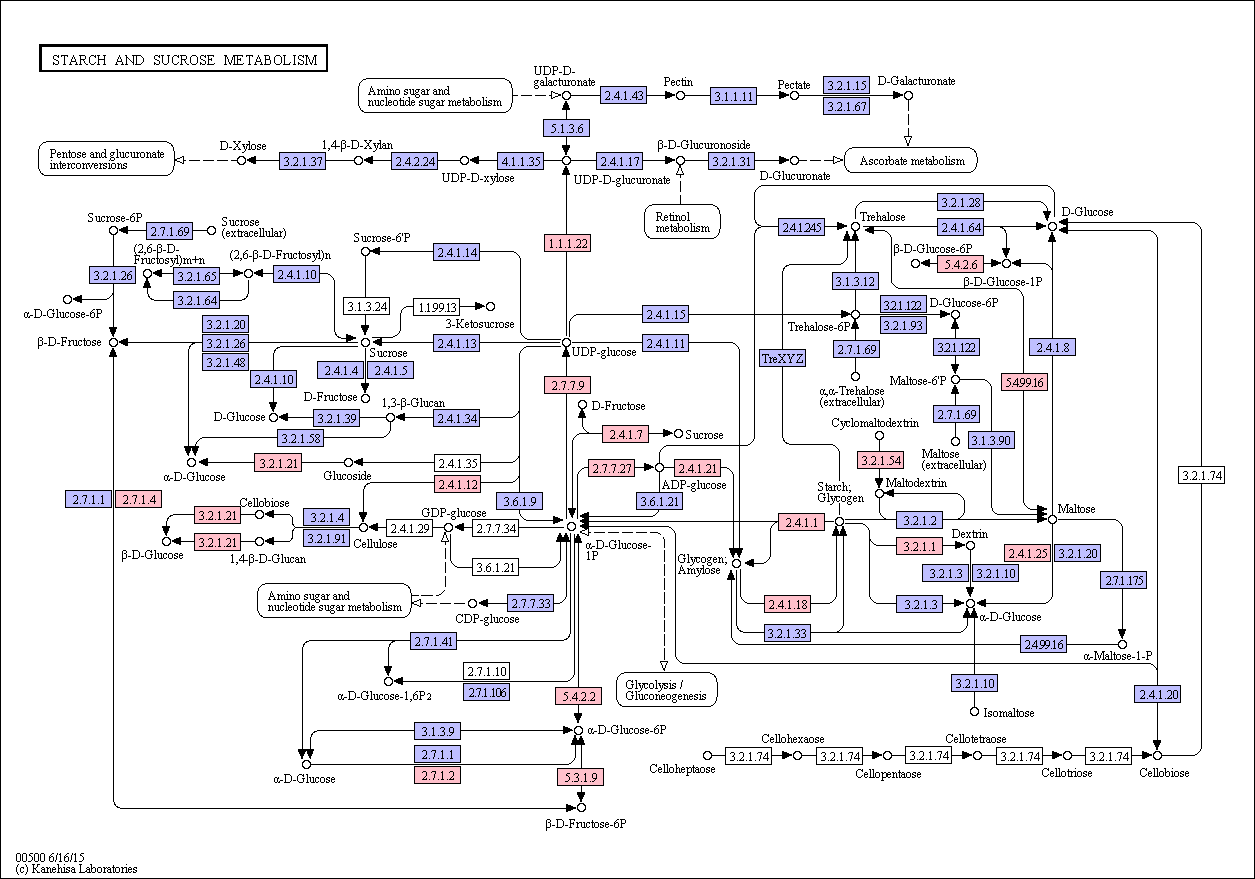

Supplement: S11 Fig — Red indicates present, blue indicates absent. (PNG) [file pone.0202792.s014.png]

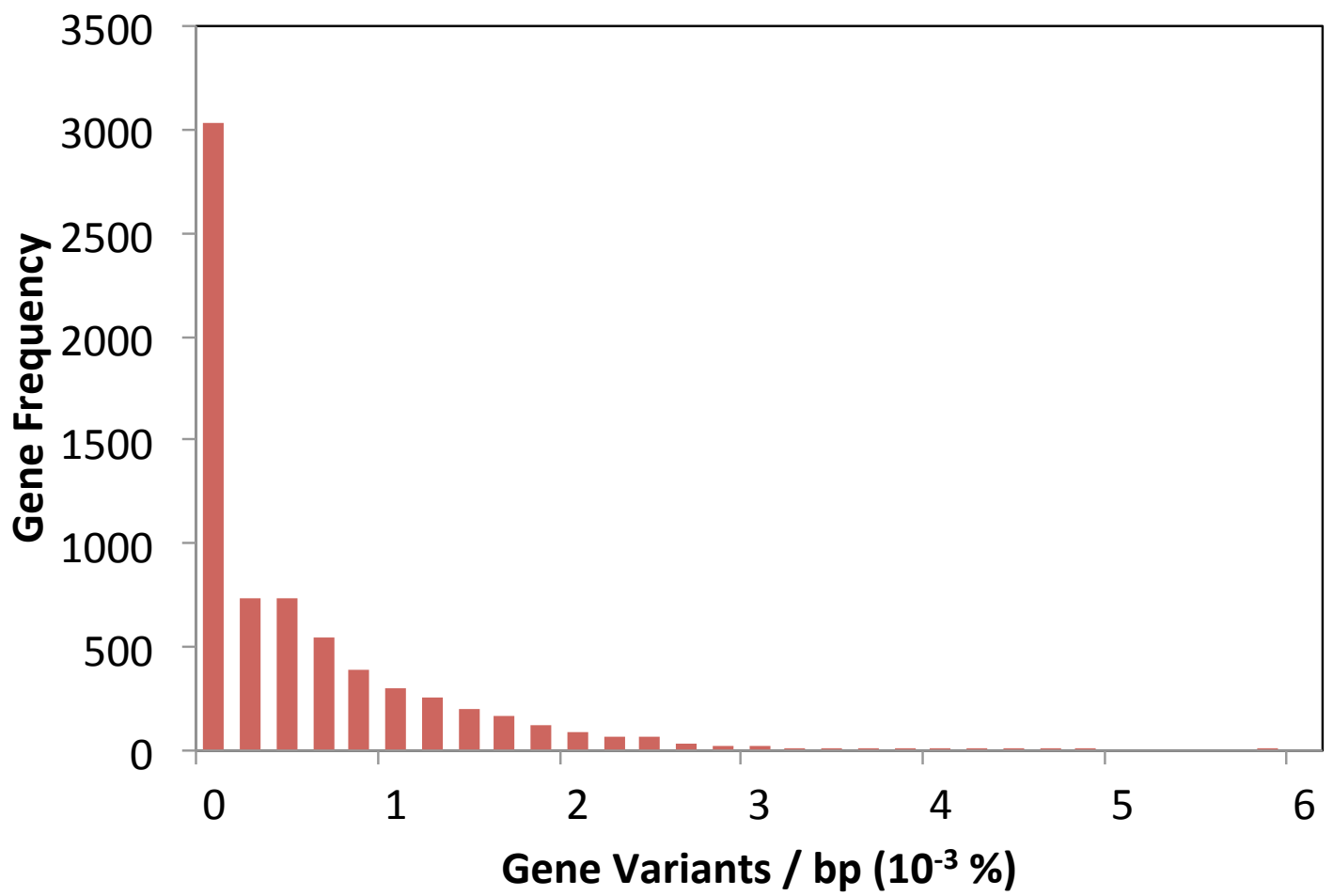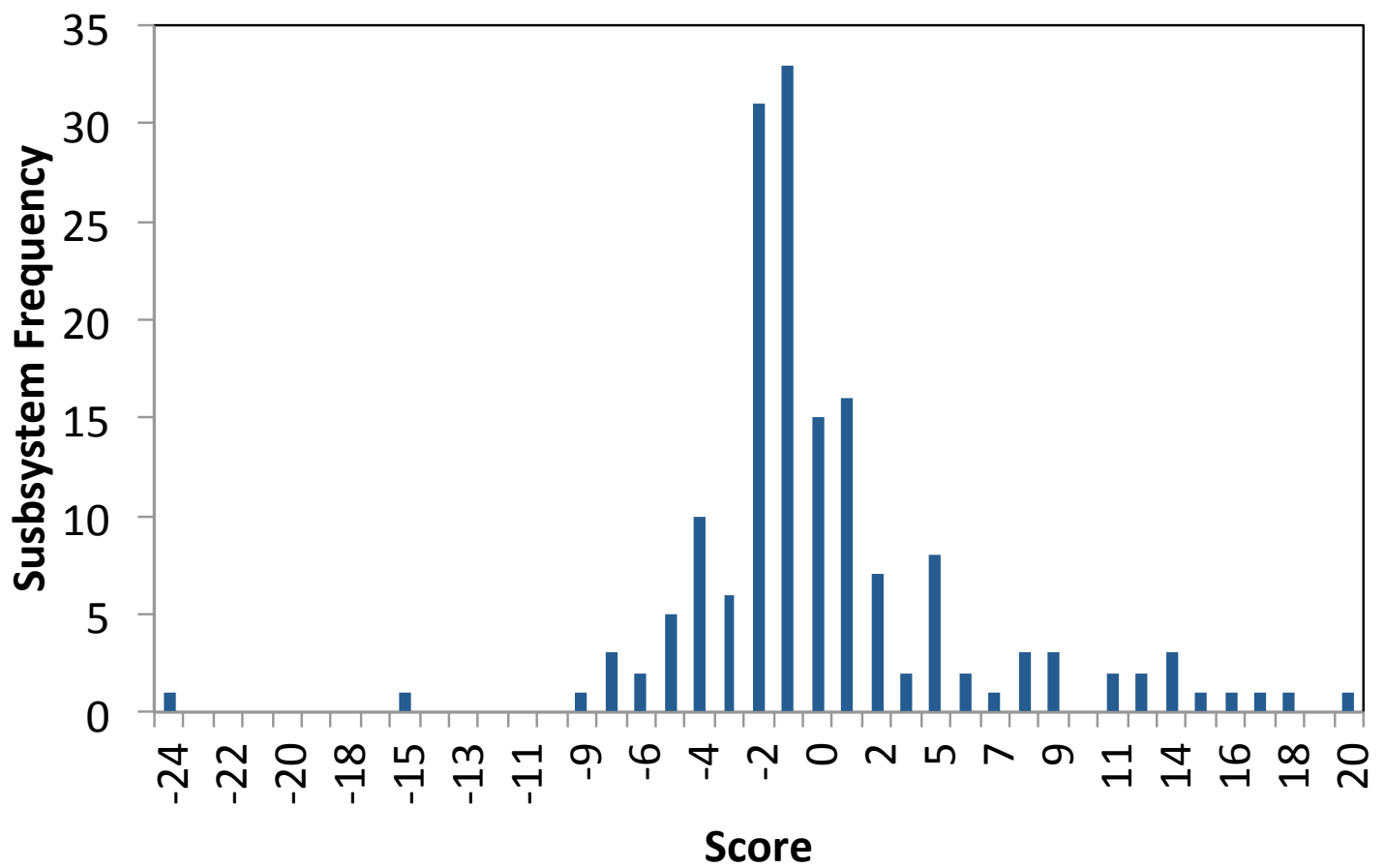

Supplement: S12 Fig — (PDF) [file pone.0202792.s015.pdf]
