# Supplementary material for: Metagenomics reveals niche partitioning within the phototrophic zone of a microbial mat
Source: PLoS One. 2018 Sep 11;13(9):e0202792. doi: 10.1371/journal.pone.0202792 (PMC6133358; doi:10.1371/journal.pone.0202792)
Supplement: S5 Fig — Binning Galaxy plots (top) and bin numbers (bottom) are paired for each assembly word size. (PDF) [file pone.0202792.s008.pdf]

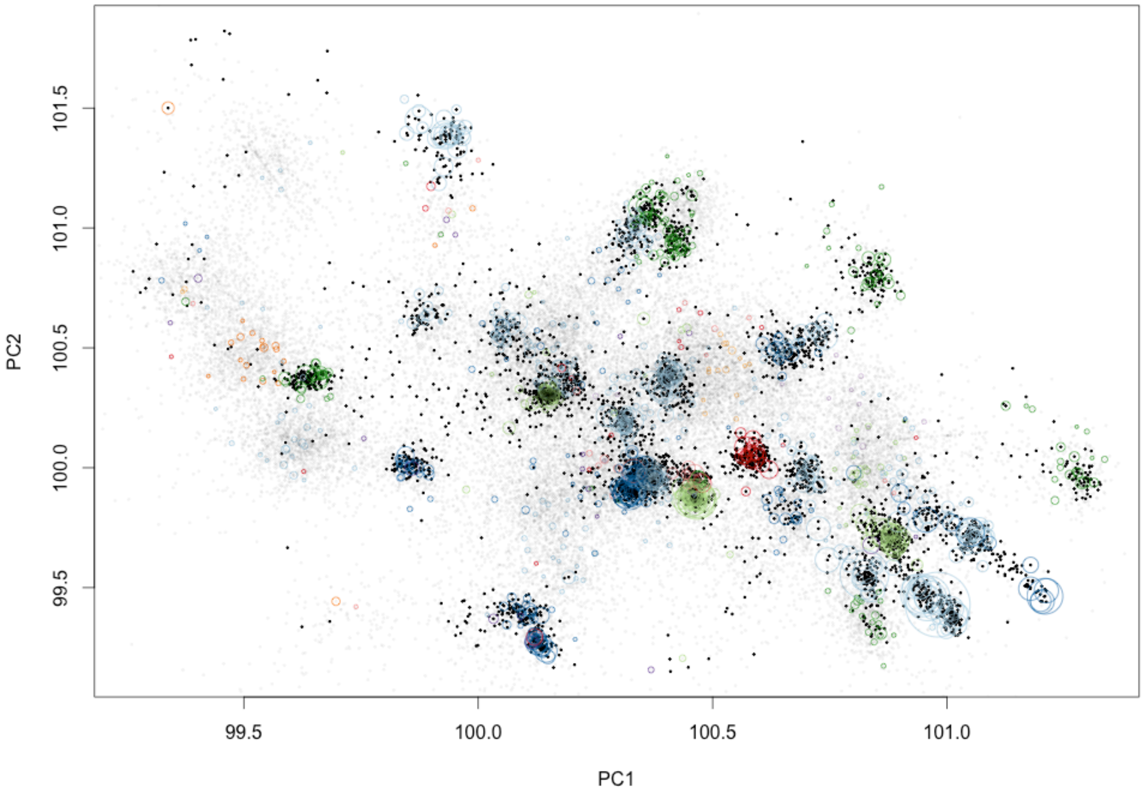
PC2
101.5
101.0
100.5
100.0
99.5
PC1
99.5
100.0
100.5
101.0

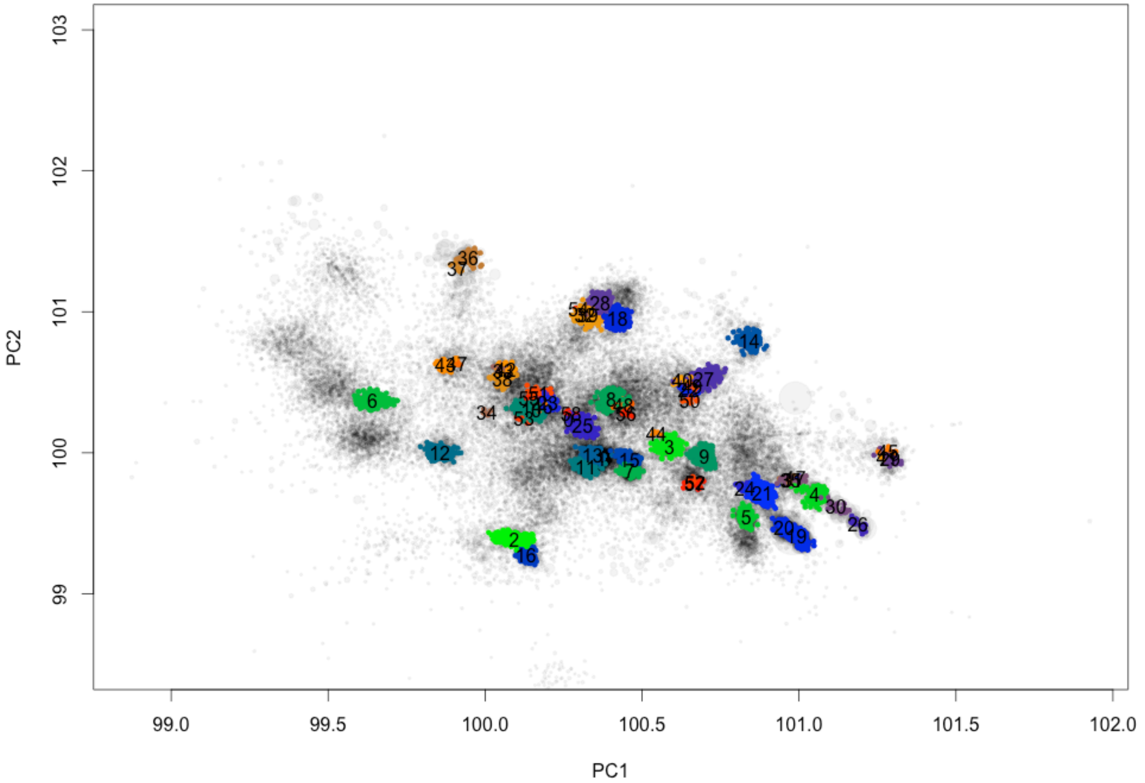
PC2
103
102
101
100
99
PC1
99.0
99.5
100.0
100.5
101.0
101.5
102.0
